# N4-acetylcytidine modifies primary microRNAs for processing in cancer cells

**DOI:** 10.1007/s00018-023-05107-w

**Published:** 2024-02-03

**Authors:** Hailong Zhang, Runhui Lu, Jiayi Huang, Lian Li, Yingting Cao, Caihu Huang, Ran Chen, Yanli Wang, Jian Huang, Xian Zhao, Jianxiu Yu

**Affiliations:** 1 Department of Biochemistry and Molecular Cell Biology, Shanghai Key Laboratory of Tumor Microenvironment and Inflammation, Shanghai Jiao Tong University School of Medicine, Shanghai, 200025 China; 2 Institute of Translational Medicine, National Center for Translational Medicine (Shanghai), Shanghai Jiao Tong University, Shanghai, 200240 China

**Keywords:** N4-acetylcytidine (ac4C), NAT10, Pri-miRNA, miRNA processing, Tumorigenesis

## Abstract

N4 acetylcytidine (ac4C) modification mainly occurs on tRNA, rRNA, and mRNA, playing an important role in the expression of genetic information. However, it is still unclear whether microRNAs have undergone ac4C modification and their potential physiological and pathological functions. In this study, we identified that NAT10/THUMPD1 acetylates primary microRNAs (pri-miRNAs) with ac4C modification. Knockdown of NAT10 suppresses and augments the expression levels of mature miRNAs and pri-miRNAs, respectively. Molecular mechanism studies found that pri-miRNA ac4C promotes the processing of pri-miRNA into precursor miRNA (pre-miRNA) by enhancing the interaction of pri-miRNA and DGCR8, thereby increasing the biogenesis of mature miRNA. Knockdown of NAT10 attenuates the oncogenic characters of lung cancer cells by regulating miRNA production in cancers. Moreover, NAT10 is highly expressed in various clinical cancers and negatively correlated with poor prognosis. Thus, our results reveal that NAT10 plays a crucial role in cancer initiation and progression by modulating pri-miRNA ac4C to affect miRNA production, which would provide an attractive therapeutic strategy for cancers.

**Supplementary Information:**

The online version contains supplementary material available at 10.1007/s00018-023-05107-w.

## Introduction

Modifications of RNAs are closely linked to the occurrence and development of diverse physiological and pathological diseases [1]. RNA modifications such as N6-methyladenosine (m6A), N1-methyladenosine (m1A), 5-methylcytosine (m5C), 5-hydroxymethylcytosine (hm5C), 2′-O-methylation (Nm), N4-acetylcytidine (ac4C), as well as isomerization of uridine to pseudouridine (Ψ) are involved in regulating a variety of cellular processes, such as precursor mRNA (pre-mRNA) processing and cleavage, RNA nuclear-cytoplasm transport, RNA stability and translation [2–4]. Compared to m6A, there is relatively less research on RNA ac4C, but it is on the rise.

ac4C mostly occurs on rRNA, tRNA and mRNA, which is catalyzed by the acetyltransferase NAT10 combining with the adaptor protein THUMPD1 or accessory box C/D snoRNAs [5–9]. ac4C of tRNA enhances codon correct reading and its stability [10]; ac4C of 18S rRNA at helices 34 and 45 promotes itself biosynthesis, ribosomal 40S subunit assemble and translation accuracy [5, 6, 11]; ac4C of mRNA plays a key role in regulating mRNA translation efficiency and stability in a position-dependent manner [8, 9]. ac4C in the coding sequences (CDS) promotes translational elongation, whereas ac4C in the 5`-untranslated regions (5`-UTR) inhibits the translational initiation through the generation of repressive structures and the direct modulation of tRNA_i_^Met^ interactions [12]. Multiple cytidines on HIV-1 transcripts are ac4C-modified by host cellular NAT10 after HIV-1 virus invades, and ac4C of HIV-1 transcripts is identified as an epitranscriptomic modification to enhance viral replication *in cis* [13]. Furthermore, genome-wide analysis identifies eukaryotic genomic DNA modified by N4-acetyldeoxycytosine (4acC) [14]. However, the contribution of RNA ac4C to the regulation of gene expression is not yet fully understood, and a complete repertoire of RNA ac4C is still not established.

Recently it has been reported that RNA ac4C is widely involved in human inflammation, metabolic diseases [15], autoimmune diseases [16] and cancers. ac4C is required for sustained NLRP3 inflammasome activation by inducing HMGB1 pathway in microglia [17]. Urinary excretion of ac4C-modified nucleotides in patients with pulmonary fibrosis, colon cancer, genitourinary cancer, ovarian cancer, and breast cancer is much higher than normal [18–22]. The level of ac4C is positively correlated with the stage of colon tumors, and the amount of ac4C in the urine of patients with colon cancer is significantly reduced after surgery [20]. Notably, NAT10 is highly expressed in a variety of cancers, including colon, liver, lung and acute myeloid leukemia [23–26]. Therefore, RNA ac4C and NAT10 have the potential to serve as effective biomarkers for tumor diagnosis, poor prognosis, and clinical therapeutic targets.

MicroRNA (miRNA) is a short single stranded RNA molecule (~ 22 nt) that is processed from a stem-loop hairpin sharped pri-miRNA transcript through the microprocessor DGCR8/DROSHA in the nucleus and further cleaved by DICER1 in the cytoplasm [27–29]. MiRNA constitutes an RNA-induced silencing complex (RISC) to regulate targeted mRNA for gene silencing [30–33]. Modifications of miRNA play essential roles in regulating miRNA processing and gene silencing efficiency. For examples, m6A [34, 35] and m7G [36] on pri-miRNA facilitate its processing. Furthermore, the position-specific 8-oxoguanine (o8G) modification in the seed region of mature miR-1 endows with the ability to modulating the activity of gene silencing [37]. In 2020, *Sas-Chen *et al*.* found that the stem-loop hairpin structure of mRNA transcripts plays a crucial role in its ac4C modification catalyzed by NAT10/THUMPD1 [9]. Interestingly, the stem-loop hairpin structure is the most typical feature of pri-miRNA transcripts. Thus, it is reasonable that the pri-miRNA transcripts may be ac4C-modified by NAT10. Here, we found that pri-miRNAs were ac4C-modified by NAT10. Pri-miRNA ac4C significantly increased the interaction between Pri-miRNA and microprocessor DGCR8/DROSHA, thereby promoting the biogenesis of mature miRNA and affecting cancer progression.

## Materials and methods

### Antibodies and reagents

Antibody against anti-ac4C (#ab252215) was from Abcam. Antibodies against anti-NAT10 (#sc-271770) and normal mouse IgG (#sc-2025) were from Santa Cruz Biotechnology. Antibodies against anti-Flag M2 (#F1804) and anti-HA (#MMS-101 P) were from Sigma-Aldrich. Antibodies against anti-GAPDH (60004-1-Ig), anti-Tubulin (#66031-1-Ig), anti-DGCR8 (#60084-1-Ig), anti-DROSHA (27958-1-AP), normal rabbit IgG (#30000-0-AP) and anti-GST (#66001-2-Ig) were from ProteinTech Group. Puromycin (#58-58-2) was from YEASEN. 3 × Flag peptide (#F4799) and Polybrene (#H9268) were from Sigma-Aldrich. ECL chemiluminescence reagent (#32106), Lipofectamine 2000 Transfection Reagent (#11668019), Chemiluminescent Nucleic Acid Detection Module (#89880), Pierce™ RNA 3' End Biotinylation Kit (#20160) and were from ThermoFisher Scientific. Protein G Plus/Protein A agarose suspension (#IP05) was from Calbiochem. Glutathione Sepharose 4B (#17-0756-01) was from GE Healthcare Life Sciences. Dynabeads MyOne Streptavidin C1 (#65001) was from Invitrogen. N4-acetylcytidine triphosphate (#HY-111815) were from MedChemExpress. ScriptMAX™ Thermo T7 Transcription Kit (#TSK-101) was from TOYOBO. Biotin-16-UTP (#11388908910) was from Roche. Primers and probes tagged with biotin were from Sangon Biotech and listed in Supplementary Table S1.

### Cell cultures

Human embryonic kidney (HEK) 293T (Cat#TCHu150), H1299 (Cat#TCHu160), A549 (Cat#TCHu150) and DU145 were cultured in Dulbecco’s modified Eagle’s medium (DMEM, Corning) containing 10% fetal calf serum (FBS, GIBCO) with 1% penicillin–streptomycin (Invitrogen) at 37 °C with 5% CO_2_. All transfections were performed by using Lipofectamine 2000 (Invitrogen).

### Plasmids

Plasmids pCMV-HA-DROSHA and Flag-pck-DGCR8 are previously described [38]. NAT10 and THUMPD1 cDNAs were subcloned to the vectors pCD513B-Flag, pET-28a or pGEX-4T-1, respectively. The NAT10 shRNA sequences were gained from Sigma and sub-cloned into the lentiviral vector pLKO.1, generating A549, H1299 and DU145 stable cell lines with the packaging plasmids pMD2G and pCMV-dR8. The primary miRNAs including pri-let-7a-3, pri-miR-21, pri-miR-9-1 and pri-miR-29b-1 were cloned into the CD513B vector. Primer sequences for plasmid construction and shRNA sequences were listed in Supplementary Table S1.

### Ac4C-RIP-Seq

The ac4C-RIP-Seq was performed as described [39]. Briefly, total RNA was extracted by TRIZOL reagent according to the manufacturer’s instructions from A549 cell lines, then 20 μg of total RNA was treated with DNase I at 37 °C for 30 min. The RNA was randomly fragmented to ~ 200 nt by using RNA fragmentation reagents, then the RNA fragments were incubated within 400 μl RIP-lysis buffer (50 mM Tris–HCl pH 7.5, 150 mM NaCl, 1% NP-40, 0.1% SDS, 1 mM DTT, 100 units/ml RNase inhibitor (Fermentas), 400 μM VRC (New England BioLabs) and Protease inhibitor cocktail) with the anti-ac4C antibody by rotating at 4 °C for 2 h, and following incubated with twice-washed Protein A/G beads (Thermo Fisher Scientific, MA, United States, 45 μl for each sample) through low-salt RIP wash buffer (50 mM Tris–HCl pH 7.5, 150 mM NaCl, 1% NP-40, 0.1% SDS, 1 mM DTT) by rotating at 4 °C for 2 h. Subsequently, the RNAs immunoprecipitated with anti-ac4C antibody were washed 5 times with low-salt RIP wash buffer. Finally, RNAs were eluted from the beads and purified with TRIZOL reagent.

### NAT10-RIP-Seq

10-cm plates of A549 stable cell lines were washed twice with ice-cold PBS for each plate and then crosslinked by 400 mJ/cm^2^ at 254 nm UV light twice with 4 ml ice-cold PBS on ice. The crosslinked cells were collected and lysed with the RIP-lysis buffer on ice for 1 h periodic perturbation. The cell lysates were then treated with RNase A (0.1 μg/ml) at room temperature for 15 min. 4.0 μg anti-NAT10 antibody were added and incubated at 4 °C for 2 h under periodic rotation. Protein A/G-agarose beads were washed (45 μl for each sample) 2 times with low-salt RIP wash buffer and then added to the antibody-cell lysates mixture for incubation at 4 °C for 2 h with periodic rotation. After the incubation, the beads were washed three times with high-salt RIP wash buffer (50 mM Tris–HCl pH 7.5, 500 mM NaCl, 1% NP-40, 0.1% SDS, 1 mM DTT) and two times with RIP low-salt wash buffer, respectively. RNAs bound to NAT10 were treated with 100 µl of elution buffer (50 mM Tris–HCl pH 7.5, 150 mM NaCl, 1% NP-40, 0.1% SDS, 1 mM EDTA pH 8.0, 5 mg/ml Proteinase K) for 30 min at 37 °C, and then extracted by TRIZOL reagent.

### Bioinformatics analyses of ac4C-RIP-Seq and NAT10-RIP-Seq

The rRNAs were removed from the ac4C modified RNA, NAT10 bound RNA and total RNAs (as an input) by using RNAs with NEBNext rRNA Depletion Kit (New England Biolabs, Inc., Massachusetts, USA). The rRNA-depleted RNAs were constructed RNA sequencing libraries by using NEBNext® Ultra™ II Directional RNA Library Prep Kit (New England Biolabs, Inc., Massachusetts, USA) according to the manufacturer’s instructions. Constructed libraries were controlled for quality by Q30 and quantified using the BioAnalyzer 2100 system (Agilent Technologies, Inc., USA), and the libraries sequencing were performed on an illumina Hiseq 4000 sequencer (Illumina, Inc., San Diego, CA, United States) with 150 bp paired-end reads. After 3`-adaptor-trimming and removing low quality reads using cutadapt software (version 1.9.3), the high quality clean of trimmed reads were aligned to the human reference genome (UCSC hg19) by Hisat2 software (version 2.0.4). Acetylated sites on RNAs and NAT10 bound RNAs (peaks) were identified by MACS2 software (version 1.4.3). High-confidence regions of ac4C modification and binding to NAT10 and were identified by stringent cutoff threshold, and then annotated with the latest UCSC RefSeq database to connect the peak information with the gene annotation. The 1000-bp flanking sequences of known pre-miRNAs were extracted as pri-miRNAs. Differentially acetylated peaks with a fold change cutoff > 2 and P < 0.0001 were identified by diffReps. Identified ac4C peaks and NAT10 binding peaks were subjected to motif enrichment analysis by DREME, and metagene ac4C and NAT10 binding distribution was characterized by R package MetaPlotR.

### High-throughput deep sequencing for RNA-Seq and miRNA-Seq

High-throughput deep sequencing for RNA-Seq and miRNA-Seq were conducted as described previously [40]. Total RNA (1 μg) extracted from A549 cells by TRIZOL reagent was used for removing the rRNAs using Ribo-Zero rRNA Removal Kits (Illumina, San Diego, CA, USA) as the manufacturer’s instructions for the following library construction. The rRNA-depleted RNAs were constructed RNA sequencing libraries by using TruSeq Stranded Total RNA Library Prep Kit (Illumina, San Diego, CA, USA) according to the manufacturer’s instructions. Constructed RNA-Seq libraries were controlled for quality and quantified using the BioAnalyzer 2100 system (Agilent Technologies, Inc., USA), and the libraries sequencing were performed on an illumina Hiseq instrument with 150 bp paired-end reads. High-quality clean reads were aligned to the reference genome (UCSC hg19) with hisat2 software (version 2.0.4). Then, guided by the Ensembl gtf gene annotation file, cuffdiff software (part of cufflinks) was used to get the gene-level FPKM (Fragments per kilobase of exon per million fragments mapped) as the expression profiles of mRNA, and fold change and p value were calculated based on FPKM, differentially expressed mRNA were identified.

For miRNA-Seq, total RNAs were extracted from A549 cells by using TRIZOL reagent. Each extracted RNA was used for the preparation of the miRNA sequencing library, which including 3′-adaptor ligation, 5′-adaptor ligation, cDNA synthesis and PCR amplification, approximately 150 bp PCR amplicons (corresponding to ~ 22 nt miRNAs) size of products were selected. Constructed miRNA-Seq libraries sequencing were denatured as single-stranded DNA molecules, captured on Illumina flow cells, amplified in situ as clusters and finally sequenced for 50 cycles on Illumina HiSeq Sequencer according to the manufacturer’s instructions.

mRNAs targeted by miR-9-5p or miR-29b-3p were predicted by miRTarBase, miRDB or Targetscan through miRWalk 2.0 web server [41]. High-throughput sequencing for ac4C-RIP-Seq, NAT10-RIP-Seq, RNA-Seq and miRNA-Seq were all done by Cloud-Seq Biotech (Shanghai, China).

### UV-CLIP-WB

The method for UV-CLIP-WB for pri-miRNA was conducted as described with modifications [34]. Briefly, 10-cm dish of indicated cell lines were washed by ice-cold PBS and then cross-linked by 400 mJ/cm^2^ at 254 nm UV light twice with 4 ml ice-cold PBS on ice. Cells were lysed with the RIP-lysis buffer at 4 °C for 1 h, and subsequently treated with RNase A. Then cell lysates were immunoprecipitated with indicated antibodies. The beads were washed three times with high-salt wash buffer and two times with low-salt wash buffe. RNAs associated with indicated proteins were tagged with biotin by Pierce™ RNA 3' End Biotinylation Kit and then determined by Western blotting analysis with streptavidin-conjugated HRP. Ac4C modification of RNAs associated with DGCR8 or DROSHA were measured by Western blotting analysis with anti-ac4C antibody.

### Northern blotting analysis

Northern blotting analysis was described previously [42]. Briefly, RNAs were fractionated by electrophoresis on a polyacrylamide-urea gel, then the RNAs were transferred to a nylon membrane (Roche) and crosslinked by 400 mJ/cm^2^ at 254 nm UV light. The membrane was pre-hybridized by North2South® Hybridization Buffer at 55 °C for 30 min, and then was hybridized with biotinylated probe targeted to mature miRNAs and U6 snRNA by using Chemiluminescent Nucleic Acid Detection Module according to manufacturer’s instructions. Finally, the signal on membrane was detected by using Amersham Imager 600 (GE) instrument. The probe sequences were listed in Supplementary Table S1.

### qRT-PCR analysis

Briefly, total RNAs extracted by TRIZOL reagent (Invitrogen) were treated with DNase I to degrade genomic DNA. Reverse transcription was performed by using PrimeScript^TM^ RT-PCR Kit (TAKARA). For miRNA detection [43], miRNA stem-loop RT primers and U6 reverse primer were used to reverse transcription. For pri-miRNA and mRNA detection, Oligo-dT and random primers were used to reverse transcription. SYBR® Green PCR Master Mix (Applied Biosystems) was used for qPCR on StepOnePlus Real-Time PCR System (Applied Biosystems). U6 snRNA was used for normalization of mature miRNA, β-Actin or GAPDH was used for normalization of pri-miRNA and mRNA. Primers used for miRNA, pri-miRNA and mRNA are listed in Supplementary Table S1.

### ac4C-RIP-qPCR and DGCR8-RIP-qPCR

Total RNA was extracted from A549 and H1299 stable cell lines by TRIZOL reagent. Then 1 μg of total RNA used as Input, and 10 μg of total RNA were incubated within 400 μl RIP-lysis buffer with the anti-ac4C antibody by rotating at 4 °C for 2 h, and following incubated with Protein A/G beads by rotating at 4 °C for 2 h. Subsequently, pri-miRNAs pulled down by anti-ac4C antibody were washed 5 times with RIP-lysis buffer and then purified with TRIZOL reagent. Finally, Input RNAs and the ac4C modification of pri-miRNAs were quantitated via qRT-PCR.

DGCR8-RIP-qPCR was conducted as described previously [40]. Briefly, Cells were lysed in RIP-lysis buffer on ice for 1 h. 1/10 of cell lysates as Input were extracted by TRIZOL reagent, the 9/10 of cell lysates were incubated with anti-DGCR8 antibody and protein A/G-agarose beads at 4 °C overnight. Pri-miRNAs bound to DGCR8 were washed with the same RIP-lysis buffer for five times and then extracted by TRIZOL reagent. RNAs were detected via qRT-PCR. Ac4C modification of pri-miRNAs and DGCR8 bound pri-miRNAs were normalized and calculated as % of Input.

### ac4C detection by dot-blot assay

ac4C detection by dot blot assay were conducted as described [8]. Total RNAs were extracted from A549 or H1299 stable cell lines and in vitro ac4C formation assays by TRIZOL reagent, the RNAs were denatured at 95 °C for 5 min and then immediately put on ice for 2 min. Equal amounts of serial-diluted RNAs were added into an N+ nylon membrane (GE Healthcare) and then crosslinked by 480 mJ/cm^2^ at 254 nm UV light. The membrane was blocked with 5% milk in 1 × PBST for 30 min at room temperature and then inducted with anti‐ac4C antibody at 4 °C overnight. After washed three times by PBST the membrane was incubated with anti-rabbit IgG HRP conjugated secondary antibody for 1 h at room temperature, the membranes were finally imaged and analyzed. Methylene blue staining was used to verified that equal amount RNAs was spotted on the membrane.

### Preparation for biotin-labeled pri-miR-9-1, pri-miR-29b-1, 18S-rRNA-45h, ac4C-pri-miR-9-1, ac4C-pri-miR-29b-1 and ac4C-18S-rRNA-45h

The DNAs containing T7 RNA polymerase promoter and pri-miR-9-1 (137nt and 413nt) or pri-miR-29b-1 (144nt) (T7-RNA-polymerase-promoter-pri-miR-9-1/-pri-miR-29b-1) and helix 45 of 18S-rRNA (50nt) (T7-RNA-polymerase-promoter-18S-rRNA-45h) were separately amplified by PCR (Supplementary Table S1) from CD513B-pri-miR-9-1, CD513B-pri-miR-29b-1 and total RNA reverse transcribed cDNA. The 18S-rRNA-45h, pri-miR-9-1 and pri-miR-29b-1 RNAs were internally tagged with biotin by using Bio-16-UTP as well as ATP, UTP, CTP and GTP through T7 RNA polymerase transcription. Meanwhile, ac4C-pri-miR-9-1, ac4C-pri-miR-29b-1 and ac4C-18S-rRNA-45h RNAs were transcribed with ATP, UTP, GTP and N4-actylcytidine triphosphate (ac4CTP) as well as internally tagged with biotin by using Bio-16-UTP through T7 RNA polymerase transcription. Finally, all the transcribed RNAs were further purified from 8 M Urea/ 15% PAGE gel.

### Protein purification

Protein purification was conducted as described [44]. The prokaryotic expression constructs pET-28a-NAT10, pET-28a-THUMPD1 and pGEX-4T-1-DGCR8 were transformed into BL21 competent cells with 0.5 mM isopropyl β-d-1-thiogalactopyranoside (IPTG) inducing for 16 h at 16 °C. For His-NAT10/-THUMPD1 purification, bacteria were lysed in lysis buffer (50 mM Tris–HCl pH 8.0, 300 mM NaCl) with sonication. The inclusion bodies were harvested by centrifugation at 13,000 g for 20 min at 4 °C. After washing twice with wash buffer (50 mM Tris–HCl pH 8.0, 300 mM NaCl and 2 M urea) and then the inclusion bodies were dissolved in lysis buffer (50 mM Tris–HCl pH 8.0, 300 mM NaCl and 6 M urea). The denatured proteins were refolded after being dialyzed with renatured buffer (50 mM Tris–HCl pH 8.0 and 150 mM NaCl). For GST-DGCR8 purification, bacteria were lysed in lysis buffer (50 mM Tris–HCl pH 8.0, 300 mM NaCl, 1% NP-40, 100 μg/ml lysozyme, protease inhibitor cocktails and 2 U/ml DNase I) with sonication. GST-DGCR8 protein was purified with Glutathione sepharose 4B beads (GE healthcare) and gradually eluted with 20 mM GSH and 10 mM GSH for 15 min within 50 mM Tris–HCl pH 7.4, 150 mM NaCl. For Flag-NAT10 and Flag-DROSHA purification, 293T cells transfected with Flag-NAT10 or Flag-DROSHA were lysed in RIPA lysis buffer and following incubated with Flag-M2 beads overnight at 4 °C. Beads were individually washed three times with high-salt wash buffer and twice with low-salt wash buffer. Then Flag-NAT10 and Flag-DROSHA proteins were purified by 3 × Flag peptide (Sigma-Aldrich).

### Electrophoretic mobility shift assay (EMSA)

Electrophoretic mobility shift assays were conducted as described previously [45]. Briefly, in vitro prepared biotin tagged ac4C or non-ac4C modification of pri-miRNAs were individually co-incubated with purified His-NAT10, His-THUMPD1 or GST-DGCR8 in 20 µl of total volume binding buffer containing 20 mM Tris–HCl pH 7.6, 5 mM MgCl_2_, 100 mM NaCl, 10% Glycerol, 2 mM DTT and 40U RNase inhibitor (Thermo). The reactions were incubated at room temperature for 1 h, then free pri-miRNAs and His-NAT10-, His-THUMPD1- or GST-DGCR8-bound pri-miRNAs were separated by native 7% polyacrylamide gels and followed by detection with streptavidin-conjugated HRP.

Denatured pri-miR-29b-1 was obtained from in vitro purified native pri-miR-29b-1 through incubation at 95 °C for 1 min to lose its advanced structure and then immediately placed on ice to avoid its renaturation (Fig. 2c). Subsequently, the same amount of native pri-miR-29b-1 and denatured pri-miR-29b-1 were separately co-incubated with or without purified gradient-increased His-NAT10 in 20 µl of total volume EMSA binding buffer, which contains 20 mM Tris–HCl pH 7.6, 5 mM MgCl_2_, 100 mM NaCl, 10% Glycerol, 2 mM DTT and 40U RNase inhibitor (Thermo). The reactions were incubated at room temperature for 1 h, then free pri-miR-29b-1 and His-NAT10 bound pri-miR-29b-1 were separated by native 7% polyacrylamide gels and followed by Northern blotting analysis with streptavidin-conjugated HRP.

### In vitro pri-miRNA processing

The in vitro pri-miRNA processing was performed as described [34, 46]. 293T cells transfected with HA-DROSHA and Flag-DGCR8 were lysed with RIP-lysis buffer. Cell lysates expressing DROSHA/DGCR8 were co-incubated with or without prepared biotin-tagged ac4C and non-ac4C modified pri-miR-29b-1 or pri-miR-9-1 within in vitro processing buffer (20 mM Tris–HCl pH 7.5, 64 mM MgCl_2_, 75 mM NaCl, 10% glycerol and RNase inhibitor) at 37 °C for 2 h, then the reaction mix were treated with 5 mg/ml Proteinase K at 37 °C 10 min, RNAs were extracted by TRIZOL reagent, pre-miRNAs cleaved from ac4C-modified and non-ac4C-modified pri-miRNAs were determined by Northern blotting analysis with streptavidin-conjugated HRP.

### In vitro ac4C assay

In vitro prepared biotin-tagged 18S-rRNA-45h (50 nt), L-pri-miR-9-1 (413 nt) and pri-miR-29b-1 (137 nt) were individually co-incubated with purified Flag-NAT10, His-THUMPD1 and Flag-NAT10/His-THUMPD1 at 37 °C for 2 h in the reaction buffer (50 mM Tris–HCl pH7.4, 150 mM NaCl, 10 mM EDTA, 5 mM MgCl_2_, 1% NP-40, 1 mM DTT, RNase inhibitor and Protease inhibitor cocktail) with or without ATP and acetyl-coA. Then the reaction mix were treated with 5 mg/ml Proteinase K at 37 °C for 10 min, subsequently, RNAs were extracted and purified by TRIZOL reagent, ac4C-modified 18S-rRNA-45h (as the positive control) and pri-miRNAs by NAT10/THUMPD1 and non-ac4C-modified RNAs were conducted by dot blot assay and following detected by anti-ac4C antibody, after stripped anti-ac4C antibody the biotin tagged 18S-rRNA-45h, L-pri-miR-9-1 and pri-miR-29b-1 were measured by streptavidin-conjugated HRP.

### RNA pull-down assay

The RNA pull-down assay was performed as described [40]. In vitro prepared biotin tagged and ac4C or non-ac4C modification of pri-miRNAs, GST-DGCR8 protein and Dynabeads™ MyOne™ Streptavidin C1 (Invitrogen) were co-incubated in RIP-lysis buffer at 4 °C overnight. Then the Dynabeads bound pri-miRNA-GST-DGCR8 complexes were washed with RIP-lysis buffer for five times, followed by immunoblotting with anti-GST.

### Soft agar colony-formation assay

The soft agar colony-forming assay was performed as previously described [47, 48]. In briefly, 2 ml complete cell culture medium containing 10% FBS with 0.6% low melting point agarose and were placed on the bottom of six-well plates as the base layer. Then 2 × 10^3^ of A549-shNAT10 and H1299-shNAT10 stable cell lines were seeded in 2 mL of complete cell culture medium containing 10% FBS with 0.35% low melting point agarose and cultured on the solidified base gel. Cells were cultured at 37 °C in 5% CO_2_ for 2–3 weeks until colonies formed. After staining with 0.005% crystal violet, the photographs of the cell colonies were taken, and the number of colonies was counted using PHOTOSHOP CC 2019 (version 20.0.0).

### Quantification and statistical analysis

All data are presented as means ± standard deviation (SD) unless otherwise indicated. Quantification was analyzed by Student’s t tests (two-tailed and unpaired) using GraphPad Prism 8 unless otherwise indicated. qRT-PCR and soft agar assays were presented as means ± s.d. or s.e.m. (*P < 0.05; **P < 0.01; ***P < 0.001; unpaired, two-tailed Student’s t test). The mean values obtained in the control and experimental groups were analyzed for significant differences. Unless stated otherwise, the experiments were not randomized and investigators were not blinded to allocation during experiments and outcome assessment.

## Results

### Pri-miRNAs are modified by ac4C

**Fig. 1:**
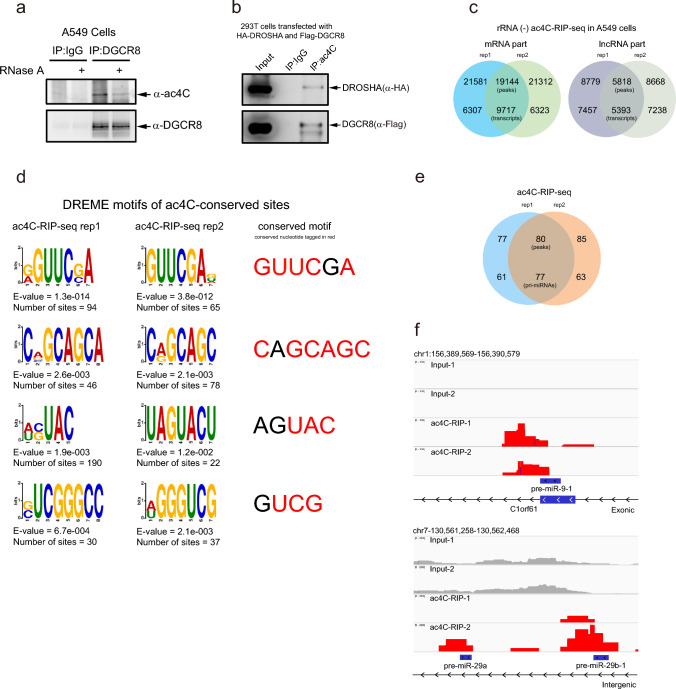
Pri-miRNAs are ac4C-modified. **a** RNAs associated with DGCR8 were ac4C-motified. A549 cells were treated by UV for cross-linking, then cell lysates were treated with RNase A and IP with anti-DGCR8 antibody. ac4C-modified RNAs bound to DGCR8 were conducted by WB with anti-ac4C antibody. **b** DROSHA and DGCR8 were pulled-down by ac4C-modified RNAs. 293T cells overexpressing with HA-DROSHA and Flag-DGCR8 were treated by UV for CLIP with anti-ac4C antibody. DROSHA and DGCR8 associated with ac4C-modified RNAs were measured by WB with indicated antibodies. **c** Overlapping of peaks and transcripts of mRNA and lncRNA identified by two replicates of rRNA (-) ac4C-RIP-Seq in A549 cells. **d** Overlapping of peaks and transcripts of pri-mRNA identified by two replicates of ac4C-RIP-Seq in A549 cells. **e** ac4C enriched motifs were identified by DREME (Discriminative Regular Expression Motif Elicitation) with ac4C-RIP-Seq peaks (E-values, the sites were found under this motif out of top 1000 scored peaks), the conserved nucleotides within these ac4C-modified motifs were colored in red. **f** IGV tracks displaying examples of sequencing read clusters from two replicates of ac4C-RIP-Seq are shown next to the pre-miR-9-1 and pre-miR-29b-1 genomic loci

To determine whether pri-miRNAs are modified by ac4C, we detected RNAs bound to the microprocessor DROSHA/DGCR8. A549 cells were treated by UV for the cross-linking immunoprecipitation (CLIP) with anti-DGCR8 antibody. Western blotting (WB) with anti-ac4C antibody showed that RNAs bound to DGCR8 were ac4C-modified, which was significantly removed by the addition of RNase A (Fig. 1a). Similarly, RNAs pull down by ectopically expressed HA-DROSHA in 293T cells were modified by ac4C, which were decreased by the treatment with RNase A (Fig. S1a). Furthermore, 293T cells transiently expressed HA-DROSHA and Flag-DGCR8 were treated with UV for the following RNA immunoprecipitation (RIP) with anti-ac4C antibody, and WB confirmed that HA-DROSHA and Flag-DGCR8 were pull down by ac4C-modified RNAs (Fig. 1b). Thus, above results illustrate that DROSHA or DGCR8-associated RNAs, mainly pri-miRNAs, are modified by ac4C.

To further verify this, ac4C-RIP-Seq was performed on rRNA-removed total RNAs extracted from A549 cells. The sequencing data showed that 19144 mRNA peaks (9717 mRNA transcripts, Supplementary Table S2) and 5818 lncRNA peaks (5393 lncRNA transcripts, Supplementary Table S3) were enriched by anti-ac4C antibody, indicating these mRNA and lncRNA transcripts were potentially modified by ac4C (Fig. 1c). We found ac4C-site clusters were mainly enriched within CDS, 3' untranslated regions (3' -UTRs) and near the translation start sites, but poorly enriched within 5'-UTR of mRNAs (Fig. S1b), which is consistent with the result from other group [8]. Furthermore, ac4C motifs were analyzed by the discriminative regular expression motif elicitation (DREME) software to show four summit motifs with GUUCGA, CAGCAGC, AGUAC and GUCG, in which ‘C’ nucleotides were modified by ac4C (Fig. 1d). Strikingly, 80 peaks in 77 pri-mRNA transcripts, (Supplementary Table S4) were identified by ac4C-RIP-Seq, indicating that these pri-mRNAs were ac4C modified (Fig. 1e). The visualization analysis by the integrative genomics viewer (IGV) software further displayed that ac4C-modified peaks completely or partially overlapped some pre-miRNAs, such as pre-miR-9-1, pre-miR-29a, pre-miR-29b-1 (Fig. 1f), pre-let-7a-1, pre-let-7f-1, pre-miR-138-1 and pre-miR-4521 (Fig. S1c). Most importantly, four sequences within both wings of pri-miR-9-1 and near the pre-miR-9-1 were predicted ac4C modified by using PACES [49] (http://www.rnanut.net/paces/, Supplementary Table S5). Therefore, above results demonstrate that pri-miRNA can undergo ac4C modification.

### NAT10 and THUMPD1 directly interact with pri-miRNAs

**Fig. 2:**
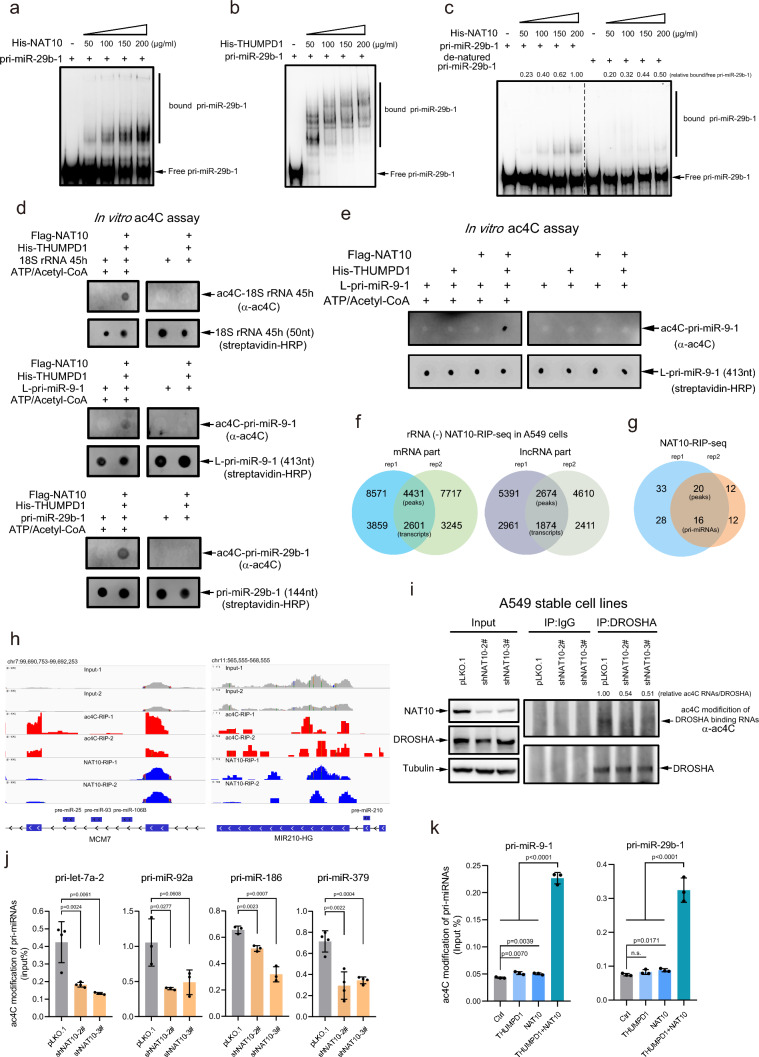
NAT10 and THUMPD1 interact with and catalyze ac4C modification of pri-miRNAs. **a-b** EMSA assays showed that NAT10 (**a**) and THUMPD1 (**b**) directly interact with pri-miR-29b-1. In vitro transcribed and biotin-tagged pri-miR-29b-1 were co-incubated with purified His-NAT10 or His-THUMPD1, then free pri-miR-29b-1 and His-NAT10- or His-THUMPD1-bound pri-miR-29b-1 were separated by native 7% polyacrylamide gels and followed by Northern blotting analysis with streptavidin-conjugated HRP. **c** EMSA assay for native and denatured pri-miR-29b-1 associated with NAT10 were performed according the Method. **d**–**e** In vitro ac4C assays for 18S-rRNA-45h, L-pri-miR-9-1 and pri-miR-29b-1. Biotin-tagged 18S-rRNA-45h, L-pri-miR-9-1 or pri-miR-29b-1 were co-incubated with purified Flag-NAT10, His-THUMPD1 or NAT10/THUMPD1 with or without ATP/acetyl-CoA. ac4C-modified RNAs and biotin-tagged RNAs were detected by dot-blotting with anti-ac4C antibody and streptavidin-conjugated HRP, respectively. **f** Overlapping of peaks and transcripts of mRNA and lncRNA identified through two replicates of rRNA (-) NAT10-RIP-Seq in A549 cells. **g** Overlapping of peaks and transcripts of pri-mRNA identified by two replicates of NAT10-RIP-Seq in A549 cells. **h** IGV tracks displaying examples of sequencing read clusters from ac4C-RIP-Seq and NAT10-RIP-Seq are shown next to the pre-miR-25/pre-miR-93/pre-miR-106B cluster and pre-miR-210 genomic loci. **i** Knockdown of NAT10 decreased the ac4C of RNAs bound to DROSHA. A549-shNAT10 stable cell lines were treated with UV and subsequently immunoprecipitated by anti-DROSHA antibody, ac4C of RNAs associated with DROSHA were immunoblotted with ac4C antibody. **j** Knockdown of NAT10 inhibited ac4C of pri-let-7a-2, pri-miR-92a, pri-miR-186 and pri-miR-379. ac4C-modified pri-miRNAs were measured by qRT-PCR. **k** Overexpression of NAT10/THUMPD1 increased ac4C of pri-miR-9-1 and pri-miR-29b-1. HEK-293T cells were transfected with NAT10, THUMPD1 and NAT10/THUMPD1, then RNAs were extracted and performed RIP with anti-ac4C antibody, and ac4C-modified pri-miRNAs were determined by qRT-PCR. Data were mean ± s.d., n >  = 3 biologically independent samples, and *P*-values were determined by unpaired two-sided t-test

Since ac4C of tRNA, rRNA and mRNA is catalyzed by NAT10 [5–11], so we speculated that it may also mediate ac4C of pri-miRNA. Firstly, to determine whether NAT10 directly interacts with pri-miRNA, purified pri-miR-9-1 (137 nt) or pri-miR-29b-1 (144 nt) from in vitro transcription mediated by T7 promoter (Fig. S2a) was co-incubated with bacterially expressed His-NAT10 or His-THUMPD1 protein (Fig. S2b) for the electrophoretic mobility shift assay (EMSA), respectively. The results showed that both NAT10 and THUMPD1 directly interacted with pri-miR-29b-1 (Fig. 2a, b) and pri-miR-9-1 (Fig. S2c, S2d). Since pri-miRNA generally contains an RNA stem-loop hairpin that is an essential secondary structure of RNA, we wanted to know whether this structure is required for the binding of pri-miRNA with NAT10. To this end, native pri-miR-29b-1 was denatured at high temperature and then immediately placed on ice to lose its secondary structure. Purified NAT10 protein was co-incubated with native pri-miR-29b-1 or denatured pri-miR-29b-1 for in vitro EMSA assay. Although the denatured pri-miR-29b-1 underwent renaturation at room temperature during the EMSA assay, the denatured pri-miR-29b-1 is only partially, but not completely, renatured. The result showed that the interaction of NAT10 with native pri-miR-29b-1 was higher than that with denatured pri-miR29b-1 (Fig. 2c). Thus, the above results suggest that NAT10 and THUMPD1 directly interact with pri-miRNA, and more importantly, the secondary structure of pri-miRNA is required for this interaction.

### NAT10/THUMPD1 catalyze ac4C of pri-miRNAs

To determine whether NAT10/THUMPD1 catalyze pri-miRNA ac4C, we performed an in vitro ac4C reaction assay. Flag-NAT10 was purified from 293T cells transiently expressing Flag-tagged NAT10 by using 3 × Flag peptide (Fig. S2e), His-THUMPD1 was purified from prokaryotic expression system (Fig. S2b), and 18S-rRNA-45h (50 nt), L-pri-miR-9-1 (413 nt; Fig. S2f), pri-miR-29b-1 (144 nt) were purified from in vitro transcription by T7 RNA polymerase (Fig. S2g). With or without ATP/Acetyl-coA, the in vitro ac4C assay showed that pri-miR9-1 and pri-miR29b-1 were ac4C-modified by NAT10/THUMPD1 (Fig. 2d), as like the positive control 18S-rRNA-45h, at the residue C1842 of which ac4C is catalyzed by NAT10/THUMPD1 [5, 6, 11]. THUMPD1 is a NAT10 adaptor for ac4C modification of tRNA [5]. In addition, *Sas-Chen *et al*.* showed that overexpression of NAT10/THUMPD1 increased ac4C of mRNAs in 293T cells, while overexpression of NAT10 or THUMPD1 alone did not [9]. To detect whether THUMPD1 is necessary in NAT10-mediated ac4C of pri-miRNAs, we performed an in vitro ac4C assay. L-pri-miR-9-1 and Flag-NAT10, His-THUMPD1, or Flag-NAT10/His-THUMPD1 were incubated with or without ATP/Acetyl-coA. The results showed that pri-miR-9-1 was ac4C-modified by NAT10/THUMPD1, but not by NAT10 or THUMPD1 alone (Fig. 2e). This result confirms that NAT10/THUMPD1 can catalyze ac4C of pri-miRNAs in vitro.

To investigate whether NAT10 is involved in pri-miRNA ac4C in cells, we conducted the high-throughput sequencing of RNAs isolated by RIP with anti-NAT10 antibody in A549 cells. 4431 mRNA peaks (2601 mRNA transcripts, Supplementary Table S6) and 2674 lncRNA peaks (1874 lncRNA transcripts, Supplementary Table S7) were pull-down by NAT10 (Fig. 2f). The enrichment motifs by NAT10 were analyzed by DREME to show that four conserved motifs, including GCUGCU, UUCUCCA, CAUCA and CCAGGA, which were specifically recognized or bound by NAT10 (Fig. S2h). We observed NAT10-site clusters were majorly enriched within CDS and 3'-UTR, but much less enriched within 5'-UTR region of mRNAs (Fig. S2i), which was identical to ac4C-enriched-site clusters (Fig. S1b). More importantly, 20 peaks in 16 pri-mRNA transcripts (Supplementary Table S8) were identified by NAT10-RIP-Seq, suggesting that a subset of pri-mRNA transcripts were bound to NAT10 (Fig. 2g). Furthermore, the density distribution showed that enriched peaks of both NAT10-RIP-Seq and ac4C-RIP-Seq were mainly located to the distal of both wings of pri-miRNAs (Fig. S2j). Furthermore, the peaks overlapped by ac4C-RIP-Seq and NAT10-RIP-Seq were analyzed by the IGV software, showing that the wings position of pri-miR-25/pri-miR-93/pri-miR-106B clusters, miR-210-HG (Fig. 2h), pri-miR-4747 and pri-miR-1178 (Fig. S2k) were simultaneously enriched with both ac4C-modified peaks and NAT10-bound peaks. Thus, the above results indicate that NAT10 catalyzes pri-miRNA ac4C in cells.

To further confirm this, NAT10 was stably knocked down by shRNA in A549 cells, in which ac4C of 18S rRNA was greatly decreased (Fig. S2l). Stable A549-shNAT10 cells were treated with UV for CLIP with anti-DROSHA antibody, showing that knockdown of NAT10 inhibited ac4C of DROSHA-bound RNAs (Fig. 2i). Moreover, total RNAs were extracted from NAT10-knockdown H1299 cell lines (Fig. S2m) for RIP with anti-ac4C antibody, and then ac4C-modified pri-miRNAs were detected by qRT-PCR. The results showed that knockdown of NAT10 significantly decreased the levels of ac4C modified pri-let-7a-2, pri-miR-92a-1, pri-miR-186 and pri-miR-379 (Fig. 2j). Moreover, lysates from 293T cells transfected with NAT10, THUMPD1 or NAT10/THUMPD1 were used for ac4C-RIP-qPCR. The results showed that the NAT10/THUMPD1 significantly augmented the ac4C levels of pri-miR-9-1 and pri-miR-29b-1, while NAT10 or THUMPD1 alone slightly increased the ac4C levels of pri-miR-9-1 and pri-miR-29b-1 (Fig. 2k). Thus, the above results demonstrate that NAT10/THUMPD1 is responsible for ac4C of pri-miRNAs.

### Knockdown of NAT10 decreases the levels of mature miRNAs

**Fig. 3:**
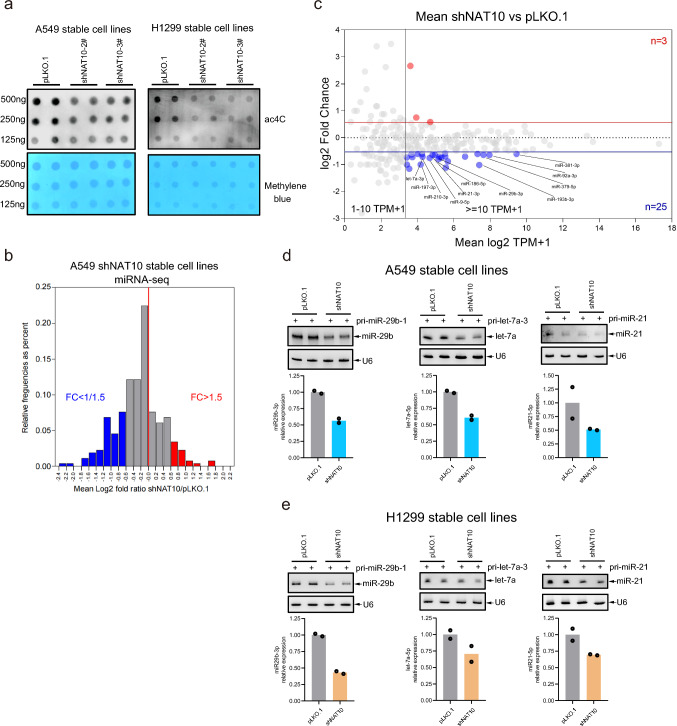
Knockdown of NAT10 decreased the expression levels of mature miRNAs. **a** Knockdown of NAT10 decreased ac4C of total RNAs in A549 and in H1299 cell lines. ac4C of RNAs were detected by dot blotting with anti-ac4C antibody, and total RNA were stained by methylene blue. **b** Histogram depicting fold change (log2) of miRNA expressions in A549-shNAT10 stable cell lines by high-throughput miRNA sequencing (miRNA-Seq), the mean ratio of shNAT10/pLKO.1 < 1/1.5 and > 1.5 were labelled with blue and red, respectively. **c** Scatter plot of miRNA expression profiles by miRNA-Seq in A549-shNAT10 stable cell lines. **d-e** Knockdown of NAT10 inhibited the expression levels of mature miR-29b, let-7a and miR-21 in A549 (**d**) and H1299 (**e**) cells. Total RNAs were extracted from A549 and H1299 cells ectopically expressing pri-miR-29b-1, pri-let-7a-3 or pri-miR-21, were determined by Northern blotting analyses for the expression levels of mature miR-29b, let-7a and miR-21. The quantification was normalized to U6 snRNA by using ImageJ

To test whether pri-miRNA ac4C is involved in regulating the biogenesis of mature miRNA, we detected the ac4C levels of total RNAs from stable cell lines A549-shNAT10 and H1299-shNAT10 by dot blotting, showing that knockdown of NAT10 globally inhibited the ac4C levels (Fig. 3a). Next, we performed the high-throughput sequencing of miRNA (miRNA-Seq, Supplementary Table S9) in A549-shNAT10. Compared with A549-pLKO.1 cells, miRNAs with 1.5 folds-change in A549-shNAT10 cells were considered to be significantly differential expressed miRNAs. The histogram showed that the proportion of down-regulated miRNAs (blue) was higher than that of up-regulated miRNA (red) (Fig. 3b). The scatter plot of miRNA expression profiles showed that the decreased miRNAs were 8.333 times more than the increased miRNAs (mean TPM + 1 >  = 10, considered as the effective candidates) (Fig. 3c). The NAT10-regulated miRNAs were shown by heatmap (Fig. S3a) and listed (Fig. S3b). These results suggested that knockdown of NAT10 reduced the expression levels of some mature miRNAs. To support this conclusion as a common phenomenon in cancer, the prostate cancer cell line DU145 was used. NAT10 was stably knocked down in DU145 (Fig. S3c) and then the expression levels of mature miRNAs, including miR-9-5p, miR-29b-3p, let-7a-3p, miR-21-3p and miR-186-5p, were determined by qRT-PCR, showing that knockdown of NAT10 reduced the expression levels of these mature miRNAs (Fig. S3d). To further verify this, pri-miR-29b-1, pri-let-7a-3 and pri-miR-21 were transiently expressed in A549-shNAT10 and H1299-shNAT10 cells, and the following Northern blotting showed that knockdown of NAT10 suppressed the expression levels of mature miR-29b, let-7a and miR-21 in A549 (Fig. 3d) and H1299 cells (Fig. 3e). Therefore, above results reveal that the expression levels of mature miRNAs are decreased when NAT10 is knocked down.

### ac4C promotes pri-miRNA processing into pre-miRNA

**Fig. 4:**
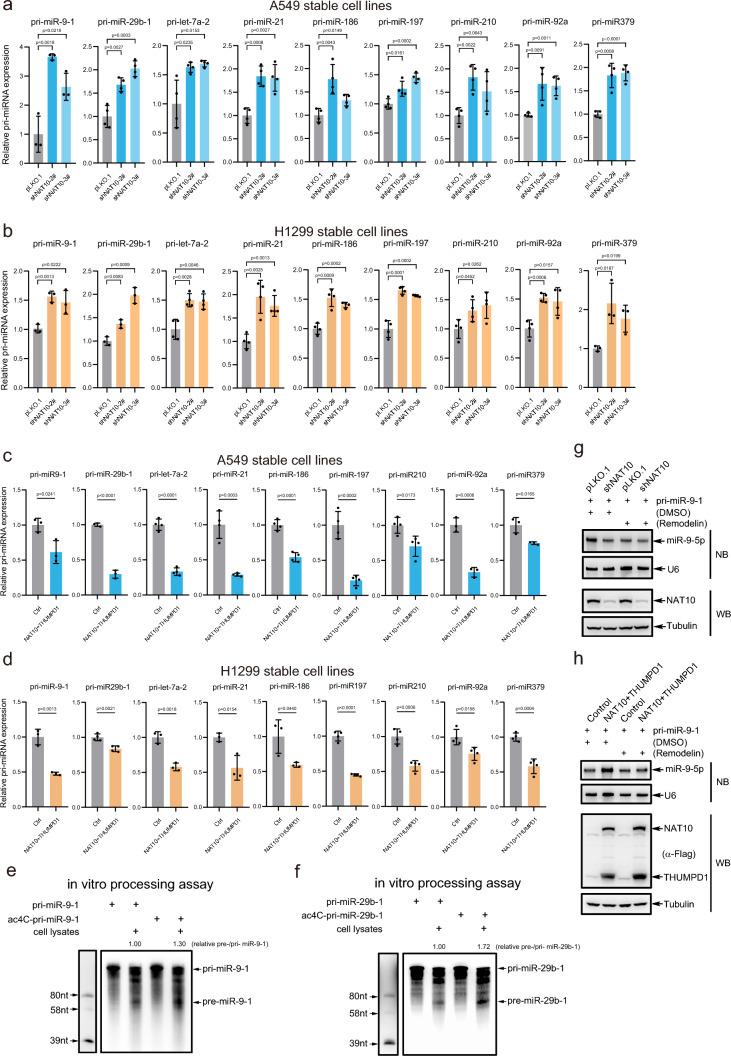
Pri-miRNA ac4C promotes its processing. **a**-**d** qRT-PCR for the expression levels of pri-miRNAs in NAT10-knockdown A549 (**a**) and H1299 (**b**) cell lines, and in NAT10/THUMPD1-overexpressed A549 (**c**) and in H1299 (**d**) cell lines. The expression levels of pri-miRNAs were determined by qRT-PCR and normalized by β-actin. Data were mean ± s.d., n >  = 3 biologically independent samples, and *P*-values were calculated by unpaired two-sided t-test. **e**-**f** ac4C of pri-miR-9-1 (**e**) and pri-miR-29b-1 (**f**) promotes their processing. Biotin-tagged ac4C- or non-ac4C- modified pri-miR-9-1 and pri-miR-29b-1 were separately co-incubated with lysates of 293T cells transfected with HA-DROSHA and Flag-DGCR8 for in vitro pri-miRNA processing assays, and then pri-miRNA and processed pre-miRNA by DROSHA/DGCR8 were determined by Northern blotting analysis through streptavidin-conjugated HRP. **g**-**h** Remodelin suppresses the biogenesis of miR-9-5p processed from pri-miR-9-1. A549-shNAT10 (**g**) and A549-Flag-NAT10/THUMPD1 (**h**) stable cell lines transfected with pri-miR-9-1 were treated with DMSO or small molecule inhibitor Remodelin, RNAs were extracted by TRIZOL reagent, the miR-9-5p and NAT10 were analyzed by Northern blotting and Western blotting, respectively

Above results proved that knockdown of NAT10 decreased the levels of mature miRNAs and pri-miRNA ac4C, so we speculated that pri-miRNA ac4C may affect the miRNA biogenesis. To verify this, we performed qRT-PCR for pri-miRNAs to show that stable knockdown of NAT10 increased the levels of pri-miR-9-1, pri-miR-29b-1, pri-let-7a-2, pri-miR-21, pri-miR-186, pri-miR-197, pri-miR-210, pri-miR-92a and pri-miR-379 in A549 (Fig. 4a) and H1299 cells (Fig. 4b). To further validate above results, pri-miRNAs in A549 and H1299 stably overexpressing NAT10/THUMPD1 (Figs. S4a, S4b) were determined by qRT-PCR to show that overexpression of NAT10/THUMPD1 decreased the levels of above pri-miRNAs A549 (Fig. 4c) and H1299 cells (Fig. 4d). Thus, these results indicate that the levels of pri-miRNAs are regulated by NAT10/THUMPD1 probably through catalyzing pri-miRNA ac4C.

When NAT10 was knocked down, the expression levels of pri-miRNA and mature miRNA were up-regulated and down-regulated, respectively, so we speculated that pri-miRNA ac4C might be involved in regulating its processing. To verify this, in vitro pri-miRNA processing assays were conducted. ac4C-modified or non-ac4C-modified pri-miR-9-1 (137 nt) and pri-miR-29b-1 (144 nt), which were purified in vitro through T7 RNA polymerase by using the material of ac4CTP or CTP along with ATP, GTP and UTP, respectively (Fig. S4c, S4d), were co-incubated with or without lysates of 293T cells transiently expressing DROSHA/DGCR8. The results showed that pre-miR-9-1 and pre-miR-29b-1 cleaved from ac4C-pri-miR-9-1 and ac4C-pri-miR-29b-1 were more than those from non-ac4C-modified pri-miR-9-1 and pri-miR-29b-1, respectively (Fig. 4e, f). Moreover, Remodelin, a small molecule inhibitor of NAT10 acetyltransferase activity [50], was used to verify the hypothesis. A549-pLKO.1 and A549-shNAT10 cells transfected pri-miR-9-1 were treated with DMSO or Remodelin. Northern blot results showed that Remodelin greatly suppressed the biogenesis of miR-9-5p in A549-pLKO.1 while having a slight effect in A549 shNAT10 cells (Fig. 4g). Similarly, we performed the experiments in A549 overexpressing NAT10/THUMPD1 cells to show that Remodelin decreased the biogenesis of miR-9-5p in A549-NAT10/THUMPD1 cells (Fig. 4h). Therefore, these results demonstrate that pri-miRNA ac4C promotes its processing.

### Pri-miRNA ac4C enhances its interaction with DGCR8

**Fig. 5:**
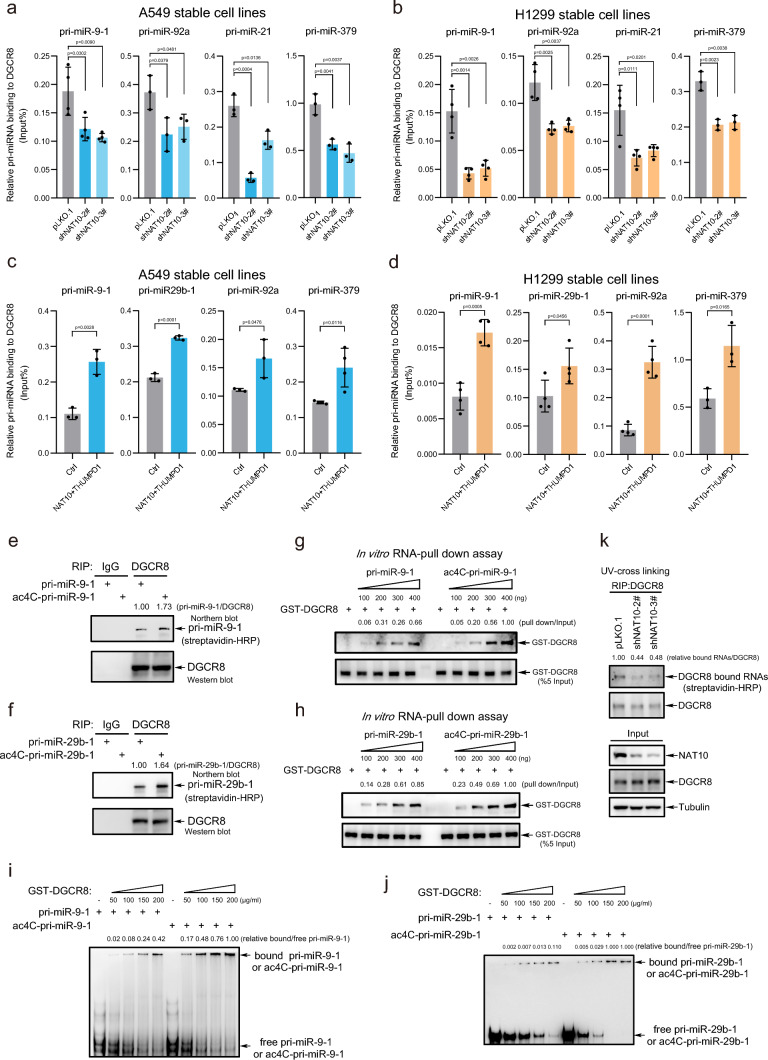
Pri-miRNA ac4C enhances its interaction with DGCR8. **a-d** RIP-qPCR for analyses of pri-miRNAs bound to DGCR8 in A549 and H1299 stable cell lines. qRT-PCR data were mean ± s.d., n >  = 3 biologically independent samples, and *P*-values were calculated by unpaired two-sided t-test. **e–f** RIP-Northern blotting/WB for analyses of ac4C-modified pri-miRNAs bound to DGCR8. A549 cell lysates were immunoprecipitated with anti-DGCR8 antibody, then beads were co-incubated with biotin-tagged pri-miR-9-1 or ac4C-pri-miR-9-1 (**e**), and pri-miR-29b-1 or ac4C-pri-miR29b-1 (**f**). RNAs bound to DGCR8 were extracted and followed by Northern blotting analysis with streptavidin-conjugated HRP. **g-h** In vitro RNA pull down assays. Biotin-tagged pri-miR-9-1 or ac4C-pri-miR-9-1 (**g**), and pri-miR-29b-1 or ac4C-pri-miR-29b-1 (**h**) were co-incubated with GST-DGCR8. GST-DGCR8 pulldown by pri-miRNAs were determined by Western blotting. **i-j** EMSA assays. Biotin-tagged pri-miR-9-1 or ac4C-pri-miR-9-1 (**i**), and pri-miR-29b-1 or ac4C-pri-miR-29b-1 (**j**) were co-incubated with GST-DGCR8. Free pri-miRNAs and protein/pri-miRNA complex were separated on native 7% polyacrylamide gels, and followed by Northern blotting analysis with streptavidin-conjugated HRP. **k** CLIP/WB assay for analysis of the amount of RNAs associated with DGCR8 in A549-shNAT10 stable cell lines. Cells were treated with UV and subsequently immunoprecipitated by anti-DGCR8 antibody, then RNAs bound to DGCR8 were tagged with biotin using Pierce™ RNA 3' End Biotinylation Kit. RNAs bound to DGCR8 were analyzed by Western blotting with streptavidin-conjugated HRP

In order to explore the molecular mechanism by which pri-miRNA ac4C enhanced its processing, we investigated whether NAT10 affects the formation of the microprocessor, DROSHA/DGCR8, which controls pri-miRNA processing into pre-miRNA. Lysates from H1299 cells with stable knockdown of NAT10 were used for co-immunoprecipitation (IP) with anti-DROSHA antibody and Western blotting, showing that knockdown of NAT10 did not affect the interaction of DROSHA with DGCR8 (Fig. S5a). We further explored whether ac4C affects the binding of pri-miRNA with DGCR8, RNA immunoprecipitation (RIP) and subsequent qRT-PCR results showed that the bindings of pri-miR-9-1, pri-miR-92a, pri-miR-21, and pri-miR-379 with DGCR8 were suppressed in A549 (Fig. 5a) and H1299 (Fig. 5b) cells with stable knockdown of NAT10. On the contrary, the bindings of pri-miR-9-1, pri-miR-92a, pri-miR-21, and pri-miR-379 with DGCR8 were enhanced in A549 (Fig. 5c) and H1299 (Fig. 5d) cells stably overexpressing NAT10/THUMPD1. These results suggest that knockdown of NAT10 reduces pri-miRNA binding to DGCR8 by inhibiting pri-miRNA ac4C.

To further verify this, endogenous DGCR8 in A549 cells were pulled down by IP, and then were co-incubated with purified pri-miR-9-1 (non-ac4C-modified) or ac4C-pri-miR-9-1, and pri-miR-29b-1 (non-ac4C-modified) or ac4C-pri-miR-29b-1 (Fig. S5b). The following Northern blotting results showed that the interactions of DGCR8 with ac4C-modified pri-miR-9-1 and pri-miR-29b-1 were stronger than those of non-ac4C-modified pri-miR-9-1 and pri-miR-29b-1 (Fig. 5e, f). Further, purified prokaryotic expressed GST-DGCR8 protein (Fig. S5c) was co-incubated with biotin-labeled pri-miR-9-1 or ac4C-pri-miR-9-1, and pri-miR-29b-1 or ac4C-pri-miR-29b-1 for RNA pull-down assays by using streptavidin-conjugated beads, and the results showed that GST-DGCR8 protein was more pulled down by ac4C-pri-miR-9-1 or ac4C-pri-miR-29b-1 than by non-ac4C-modified pri-miR-9-1 or pri-miR-29b-1, respectively (Fig. 5g, h). Similarly, ac4C-modified or non-ac4C-modified pri-miR-9-1 and pri-miR-29b-1 were incubated with GST-DGCR8 protein for EMSA assays, respectively. The results showed that DGCR8 bound more to ac4C-pri-miR-9-1 or ac4C-pri-miR-29b-1 than DGCR8 bound to non-ac4C-modified pri-miR-9-1 or pri-miR-29b-1, respectively (Fig. 5i, j). In addition, A549-shNAT10 stable cell lines were treated with UV for CLIP with anti-DGCR8 antibody, and RNAs (mainly pri-miRNAs) bound to DGCR8 were labeled with biotin and detected by Western blotting, showing that knockdown of NAT10 reduced the level of DGCR8-recruited RNAs (Fig. 5k). Therefore, above data illustrate that pri-miRNA ac4C promotes its association with DGCR8.

To explore whether NAT10 interacts with DGCR8 on pri-miRNAs, A549 cells were lysed with RIPA-lysis buffer and then treated with or without RNase A to eliminate RNAs. Then, cell lysates were immunoprecipitated with anti-NAT10 antibody for Western blotting analysis, showing that NAT10 could bind to DGCR8, although the amount of NAT10 was decreased by RNase A, the binding of DGCR8 with NAT10 was not changed and even increased. This result indicated that the interaction between NAT10 and DGCR8 seemed to be independent on pri-miRNAs (Fig. S5d). To further explore whether NAT10 affects the interaction between DGCR8 and pri-miRNAs, or vice versa, whether DGCR8 affects the interaction between NAT10 and pri-miRNAs. Purified GST-DGCR8, His-NAT10 and L-pri-miR-9-1 were co-incubated in vitro conditions, and then pulled down by GST-beads or RIP with anti-NAT10 antibody, the levels of L-pri-miR-9-1 binding to GST-DGCR8 or NAT10 was extracted and detected by qRT-PCR (Fig. S5e). GST pull-down assays showed that L-pri-miR-9-1 directly interacted with GST-DGCR8, and NAT10 could significantly reduce the interaction of L-pri-miR-9-1 with GST-DGCR8 (Fig. S5f). Similarly, NAT10-RIP showed that L-pri-miR-9-1 directly interacted with His-NAT10, and GST-DGCR8 decreased the association of L-pri-miR-9-1 with NAT10 (Fig. S5g). Therefore, these results demonstrated that DGCR8 and NAT10 competitively interacted with pri-miRNAs.

In order to verify whether DROSHA interacts directly with ac4C-modified pri-miRNAs or it is only through DGCR8, we purified Flag-tagged DROSHA in 293T cells transiently transfected with Flag-DROSHA by using 3 × Flag peptide (Fig. S5h). Purified ac4C-pri-miR-9-1 or ac4C-pri-miR-29b-1 was co-incubated with purified GST-DGCR8 or Flag-DROSHA in vitro conditions for EMSA assays. The results showed that ac4C-pri-miR-9-1 and ac4C-pri-miR-29b-1 interacted with only DGCR8, but not with DROSHA (Figs. S5i, S5j). These results were consistent with other group`s result of that DROSHA associated to pri-miRNAs through DGCR8, and once DGCR8 lacks any RNA-binding regions, the affinity of the microprocessor complex to pri-miRNAs will be lost [51]. Therefore, our results propose a model that non-ac4C modified pri-miRNAs are conventionally processed by the microprocessor DGCR8/DROSHA; Whereas, a subset of pri-miRNAs are first bound and modified with ac4C by NAT10/THUMPD1, which enhances the affinity of pri-miRNAs for binding to DGCR8, thereby promoting the processing of these pri-miRNAs into mature miRNAs (Fig. S5k).

### NAT10 is highly expressed in cancers and negatively correlated with poor prognosis

**Fig. 6:**
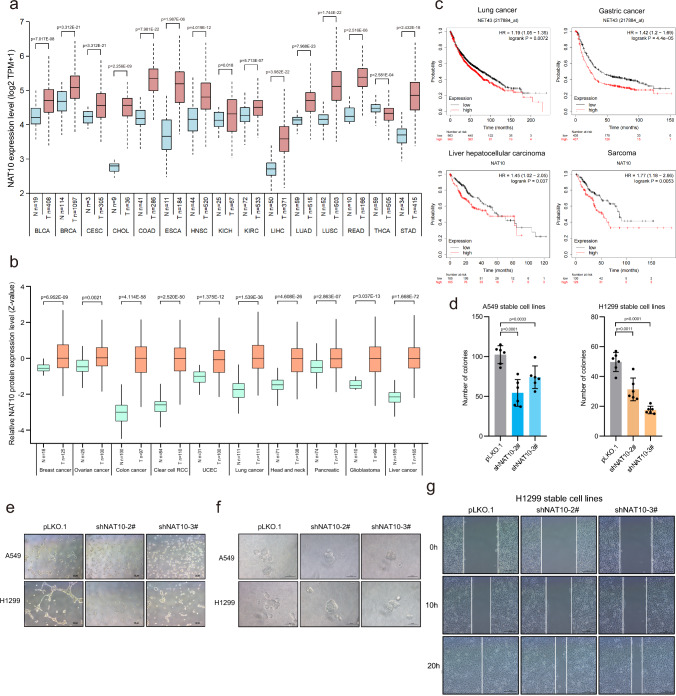
NAT10 is highly expressed in cancers and negatively correlated with poor prognosis. **a** The expression levels of *NAT10* mRNA in different tumor types or specific tumor subtypes from TCGA database. The expression levels of *NAT10* mRNA in various tumor tissues were more than those in normal tissues. **b** The expression levels of NAT10 protein in different tumor types from CPTAC database. Data were mean ± s.d., and *P*-values were determined by unpaired two-sided t-test (**a-b**). **c** The correlations between the NAT10 expression level and the survival of lung cancer, gastric cancer, liver hepatocellular carcinoma or sarcoma patients were analyzed by the Kaplan–Meier method. **d** Knockdown of NAT10 inhibited the soft agar colony formation. A549-/ H1299-shNAT10 stable cell lines were employed for anchorage-independent growth assay. Viable colonies after about 3 weeks were counted. The data of colony numbers were mean ± s.d., n = 6 biologically independent samples, and *P*-values were determined by unpaired two-sided t-test. **e–g** Knockdown of NAT10 decreased cell invasion through vasculogenic mimicry assay (**e**), 3D cell culture growth assay (**f**) and cell migration by wound-healing assay (**g**)

The abnormal expression and dysfunction of miRNA are closely related to human diseases, such as metabolic disorders, autoimmune diseases, and tumorigenesis [52]. Since above results proved that NAT10 was a key regulator in miRNA biogenesis through mediating pri-miRNA ac4C, so we wanted to know whether NAT10 plays key roles in tumorigenesis through NAT10-regulated miRNAs. Firstly, we analyzed *NAT10* mRNA expression data from the TCGA database (The Cancer Genome Atlas, https://portal.gdc.cancer.gov/) to show that the expression levels of *NAT10* mRNA in various tumors were much higher than those in normal tissues. The expression levels of *NAT10* mRNA in cholangiocarcinoma (CHOL), colon cancer (COAD), esophageal cancer (ESCA), head and neck squamous cell carcinoma (HNSC), liver cancer (LIHC), lung squamous cell carcinoma (LUSC), rectal adenocarcinoma (READ) and stomach adenocarcinoma (STAD) were significantly higher than those in normal (Fig. 6a), while the non-significant (p > 0.05) cancer types have been shown in the Fig. S6a. We also analyzed the NAT10 protein expression data from the CPTAC protein database (Clinical Proteomic Tumor Analysis Consortium, https://cptac-data-portal.georgetown.edu/). The expression levels of NAT10 protein in breast cancer, ovarian cancer, colon cancer, renal clear cell carcinoma, endometrial carcinoma, lung cancer, head and neck squamous cell carcinoma, pancreatic cancer, glioblastoma and liver cancer were greatly higher compared to those in normal tissues (Fig. 6b). Moreover, we observed that 1.73% prevalence of NAT10 mutations (9/517) in LUAD, 1.23% (6/485) in LUSC and 1.35% (138/10156) in all cancer types, respectively (Fig S6b). We performed the survival analysis by Kaplan–Meier-plot (http://kmplot.com/analysis/) to show that higher expression levels of NAT10 had shorter survival lifetime in lung cancer (*P* = 0.0072), gastric cancer (*P* = 4.4E-05), liver cancer (*P* = 0.037) and sarcoma (*P* = 0.0053) patients (Fig. 6c). Thus, above results demonstrate that the high expression of NAT10 is associated with poor prognosis in cancers. Moreover, to determine whether NAT10 affects cancer progression, we performed a series of experiments to show that knockdown of NAT10 suppressed the soft-agar colony formation (Fig. 6d), vascular mimicry (VM) (Fig. 6e), 3D culture growth (Fig. 6f) and migration of wound healing (Fig. 6g) in A549 and H1299 cells, suggesting that NAT10 promotes cancer cell progression.

### NAT10 functions by regulating miRNA production in cancers

**Fig. 7:**
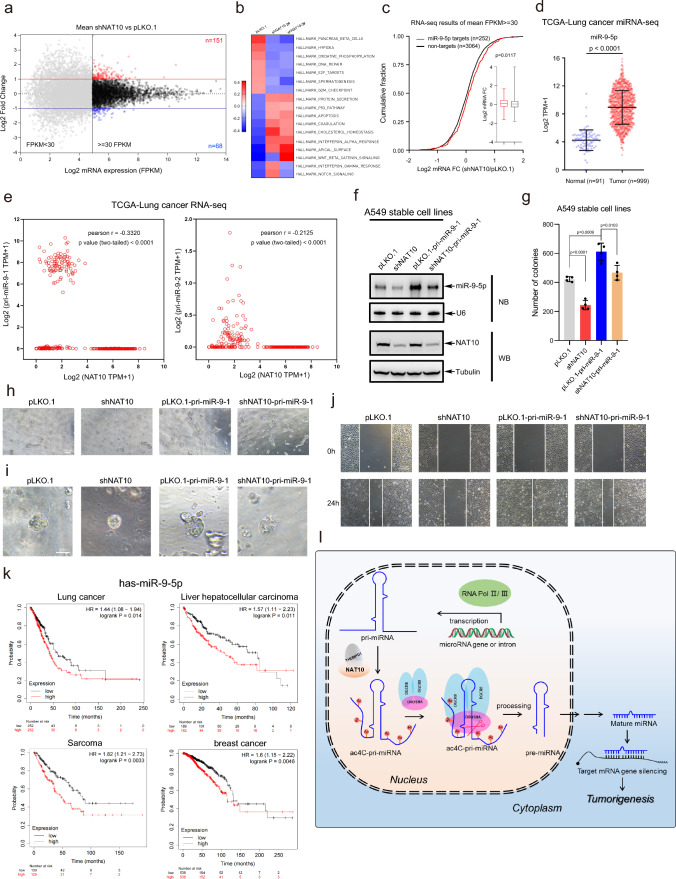
NAT10 plays oncogenic roles by regulating mature miRNA biogenesis in cancers. **a** Scatter plot of mRNA expression profiles by RNA-Seq in A549-shNAT10 stable cell lines. **b** Knockdown of NAT10 regulated the expression levels of hallmark genes, such as DNA repair, G2M checkpoint, p53 pathway and apoptosis. **c** Knockdown of NAT10 increased the accumulation of mRNA transcripts (mean FPKM >  = 30) targeted by miR-9-5p (*P* = 0.0117). Cumulative fraction analyses were performed with RNA-Seq data in A549-shNAT10 stable cell lines in which mean expression were FPKM >  = 30. In box plots, the lines represent the median, first and third quartiles, the whiskers denote the minima and maxima; *P*-values were calculated using a two-sided Mann–Whitney U test for cumulative fraction analysis. **d** Analysis of the expression level of miR-9-5p between normal tissues (n = 91) and lung cancer tissues (n = 999) from TCGA lung cancer miRNA-Seq data (*P*-value < 0.0001). Data were mean ± s.d., and *P*-values were determined by unpaired two-sided t-test. **e** The expression levels of NAT10 were negatively correlated with pri-miR-9-1 (Pearson *r* = -0.3320, *P*-value (two tailed) < 0.0001) and pri-miR-9-2 (Pearson *r* = -0.2125, *P*-value (two tailed) < 0.0001) from TCGA-Lung cancer (n = 1028) RNA-Seq data. Pearson’s correlations were analyzed by using GraphPad Prism 8. **f** Pri-miR-9-1 was stably overexpressed in A549-pLKO.1 and A549-shNAT10 cell lines. The expression of NAT10 was examined by Western blotting and miR-9-5p was determined by Northern blotting analysis. **g** miR-9-5p partially rescued the inhibition of soft agar colony formation upon NAT10 knocked down. A549 stable cell lines were employed for anchorage-independent growth assay. Viable colonies after about 3 weeks were counted. The data of colony numbers were mean ± s.d., n = 4 biologically independent samples, and P-values were determined by unpaired two-sided t-test. **h**-**j** Overexpression of pri-miR-9-1 in A549-shNAT10 cells recovered the phenotypes including vascular mimicry (**h**), 3D culture growth (**i**) and migration of wound healing (**j**), all of which were inhibited by knockdown of NAT10 in A549 cells. **k** The correlations between miR-9-5p expression level and the survival of lung cancer, liver hepatocellular carcinoma, sarcoma or breast cancer patients were analyzed by the Kaplan–Meier method. **l** A proposed model that ac4C modification of pri-miRNA promotes cancer progression by regulating mature miRNA biogenesis. Pri-miRNAs were recognized and ac4C-modified by NAT10/THUMPD1, then ac4C of pri-miRNAs enhanced its affinity to DGCR8, thereby increasing pri-miRNA processing and mature miRNA production, which ultimately affected tumorigenesis and cancer progression

To investigate NAT10-regulated miRNA downstream targets which drive cancer progression, RNA-Seq was conducted in A549 cells with stable knockdown of NAT10 to identify the mRNA transcript expression profile affected by NAT10 (Supplementary Table S10). Transcripts whose FPKM (Fragments per kilobase of exon per million fragments) > 1 presented in two samples (shNAT10-2# and shNAT10-3#) were analyzed in parallel, to reveal that knockdown of NAT10 up-regulated 151 (mean FPKM >  = 30 is considered as the effective candidates, log2 FC >  = 1) and down-regulated 68 (mean FPKM >  = 30 is considered as the effective candidates, log2 FC <  =  – 1) transcripts, respectively (Fig. 7a, Supplementary Table S10). Feature gene sets analyses showed that knockdown of NAT10 suppressed the expression levels of multiple characteristic gene sets, such as hypoxia, DNA repair, E2F targets, spermatogenesis and G2M checkpoint, while increased the expression levels of several characteristic gene sets, such as p53 pathway, apoptosis, cholesterol homeostasis and interferon alpha response (Fig. 7b). More importantly, we found that knockdown of NAT10 increased the abundance of mRNAs which targeted by NAT10-regulated miR-9-5p (*P* = 0.0117) (Fig. 7c) through cumulative fraction analyses. As expectedly, qRT-PCR results showed that knockdown of NAT10 increased the expression levels of miR-9-5p targets, including SH3BP4, NCOR2, LMNA, EPAS1 and TES (Fig. S7a). Furthermore, we analyzed data from TCGA database to show that the expression levels of mature miR-9-5p in lung cancer tissues (n = 999) were much higher than those in normal tissues (n = 91) (Fig. 7d), indicating that the high levels of NAT10 in cancers (Fig. 6a, b) up-regulated mature miR-9-5p production. Indeed, the expression levels of pri-miR-9-1 and pri-miR-9-2 were negatively correlated with the expression levels of NAT10 according to analyses of TCGA-Lung cancer RNA-Seq data (Fig. 7e).

To further verify that pri-miRNA ac4C catalyzed by NAT10 plays functional roles in cancer, pri-miR-9-1 was stably overexpressed in A549-pLKO.1 and A549-shNAT10 cells. The results showed knockdown of NAT10 decreased the expression levels of mature miR-9-5p, while the expression levels of miR-9-5p were highly and moderately expressed in A549-pLKO.1-pri-miR-9-1 and A549-shNAT10-pri-miR-9-1 cell lines, respectively (Fig. 7f). Furthermore, cell phenotypes showed that overexpression of pri-miR-9-1 in A549-shNAT10 cells recovered the phenotypes including soft-agar colony formation (Fig. 7g), vascular mimicry (Fig. 7h), 3D culture growth (Fig. 7i) and migration of wound healing (Fig. 7j), all of which were inhibited by knockdown of NAT10 in A549 cells. Taken together, these results demonstrated that NAT10 functions by regulating miRNA production in cancers.

Furthermore, the survival analysis by Kaplan–Meier-plot showed that higher expression levels of miR-9-5p in lung cancer (*P* = 0.014), liver hepatocellular carcinoma (*P* = 0.011), sarcoma (*P* = 0.0033) and breast cancer (*P* = 0.0046) patients had shorter survival lifetime (Fig. 7k), which were consistent with NAT10 pattern (Fig. 6c). In addition, very similar as miR-9-5p, the relationship of mature miR-29b-3p/pri-miR-29b-1 and NAT10 in lung cancer was obtained. Although the *P* value between miR-29b-3p targets and non-targets was not statistically significant through our RNA-seq data (p = 0.064) (Fig. S7b), which may be caused by much lesser number of miR-29b-3p targets (130) than that of non-targets (3186). However, compared to A549-pLKO.1 cells, the median expression levels of miR-29b-3p targets were higher than those of non-targets in A549-shNAT10 cells (Fig. S7b). Furthermore, the expression levels of mature miR-29b-3p in lung cancer tissues were higher than in TCGA normal tissues (Fig. S7c), and the expression levels of pri-miR-29b-1 was also negatively correlated with NAT10 in TCGA-Lung cancer RNA-Seq data (Fig. S7d). In addition, to reveal the underlying relationship between NAT10 and miRNA biogenesis, we analyzed the relationship between NAT10 with miRNA processing machineries [53] in clinical TCGA lung cancer tissues, and showed that NAT10 was positively correlated with DGCR8, DROSHA, XPO5, DICER1, AGO2 and TARBP2 (Spearman correlation coefficient (R) values of between 0.36 and 0.67) (Fig. S7e). We also found that miRNA processing machineries were positively correlated with NAT10 almost across all TCGA cancer types (Fig. S7f). Therefore, these results supported our findings that NAT10-mediated ac4C promoted its pri-miRNA processing and miRNA production in cancers.

## Discussion

NAT10 as an acetyltransferase can acetylate many protein substrates, such as p53, MORC2 and Eg5 [54–56], but also target RNAs including tRNA, rRNA and mRNA for ac4C modification with the help of its adaptor protein THUMPD1 or snoRNA. Most recently, it has been reported lncRNA is also ac4C-modified [57]. However, it is still unclear whether miRNAs have undergone ac4C modification and their potential physiological and pathological functions. In this study, we found that pri-miRNA transcripts are ac4C-modified by acetyltransferase NAT10. Knockdown of NAT10 reduced the level of pri-miRNA ac4C and subsequently inhibited the interplay of pri-miRNA with DGCR8, thereby inhibiting the processing of pri-miRNA into pre-miRNA by the microprocessor DROSHA/DGCR8. This led to the accumulation of pri-miRNA and the reduction of mature miRNA, ultimately regulating cancer progression (Fig. 7l). Thus, our findings provided new mechanistic insight into the ac4C modification modulating tumorigenesis through the miRNA pathway.

Methylations of pri-miRNAs, including METLL3-mediated m6A [34, 35] and METLL1-mediated m7G [36], are important for its processing. Inosine decoration of a subset of pri-miRNAs by RNA editing enzyme ADAR also directly regulates the processing of pri-miRNA and expression of mature miRNAs [58, 59]. Moreover, o8G of miR-1 is essential for its gene silencing efficiency [37]. In this study, we found that NAT10 and THUMPD1 directly bound to and catalyzed pri-miRNA ac4C, which promoted processing of pri-miRNA mediated by DGCR8/DROSHA. Moreover, a large number of enriched lncRNA peaks were identified to be ac4C-modified by ac4C-RIP-Seq and NAT10-RIP-Seq in our study (Figs. 1c, 2f), and further studies on the identification and biological function of lncRNA ac4C should be conducted.

The advanced structure of pri-miRNA was essential for its recognition with NAT10 for catalyzing pri-miRNA ac4C (Fig. 2c). As reported that DGCR8 directly interacts with the stem structure of pri-miRNA [28, 51, 53, 60, 61], but it is unclear whether the multivariate and advanced structure of two long arms (or called as ‘wings’) of pri-miRNA are involved in regulating the interaction between pri-miRNA and DGCR8. Here we found that ac4C occurred on both wings of pri-miRNA facilitating its association with DGCR8 for the following processing by DROSHA, however the underlying mechanism is not understood and should be further explored.

Some non-coding RNAs participate in the regulation ac4C through the interplay with NAT10. A heart-apoptosis-associated piRNA (HAAPIR) directly interacts with NAT10 to enhance its acetyltransferase activity, thereby increasing ac4C of *Tfec* mRNA transcript [39]. Long non-coding RNA LINC00623 interacts with NAT10 to block the ubiquitination-dependent degradation of NAT10 by recruiting the deubiquitinase USP39 [62]. In addition, lysine 2-hydroxyisobutyrylation (Khib) of NAT10 at lysine 823 in NAT10 enhances its interaction with USP39, resulting in increased NAT10 protein stability [63].

NAT10 plays an essential role in regulating tumorigenesis, including gastric cancer [64], colorectal cancer [23] and breast cancer [55]. We found that the expression levels of both NAT10 mRNA and protein in various tumors were significantly higher than those in normal tissue, and high expression of NAT10 was positively associated with a poor prognosis in tumor patients. NAT10 promoted tumor cell migration and malignant transformation. Therefore, NAT10 is an oncogenic protein with carcinogenic properties and can be used as a biomarker for tumor diagnosis. Development of small molecule inhibitors directly targeting NAT10 or impeding NAT10-THUMPD1 interaction could be a good strategy for tumor therapy. Interestingly, small molecule compounds targeting NAT10 K823Khib have recently been developed for anti-tumor metastasis [63].

Pri-miRNA ac4C catalyzed by NAT10 exerted its biological function in clinical cancers. Here, we found that the expression levels of NAT10-regulated mature miR-9-5p (Fig. 7d) and miR-29-3p (Fig. S7c) were highly expressed in clinical TCGA-Lung cancers compared with normal tissues. Consistently, the expression levels of pri-miR-9-1/-2 (Fig. 7e) and pri-miR-29b-1 (Fig. S7d) were negatively correlated with NAT10 in clinical TCGA-lung cancers. Among various cancer patients, higher expression levels of miR-9-5p regulated by NAT10 had a shorter survival lifetime (Fig. 7k). Our RNA-Seq data in A549-shNAT10 stable cell lines revealed that NAT10 promoted the expression of DNA repair and G2M checkpoint characteristic gene sets, while suppressed the expression of p53 pathway, apoptosis and interferon alpha response characteristic gene sets. These gene sets may also be targeted by high levels of miRNAs, such as miR-9-5p, which processed from ac4C-modified pri-miRNA. More importantly, we found that knockdown of NAT10 increased the expression levels of miR-9-5p targets, including SH3BP4, NCOR2, LMNA, EPAS1 and TES (NEW Fig. S7a), which exhibit tumor suppressive properties [65–68]. Therefore, we believe that some miR-9-5p targets may play a role in regulating tumorigenesis after NAT10 knockdown. Taken together, the outcome of this study will provide new mechanistic insight into the ac4C modification of pri-miRNAs mediated by NAT10 and useful clues for the development of novel systemic therapies and prognostic biomarkers.

### Supplementary Information

Below is the link to the electronic supplementary material.

Supplementary file1 (PDF 12798 KB)

Supplementary file2 (DOCX 20 KB)

Supplementary file3 (XLSX 9341 KB)

Supplementary file4 (XLSX 5310 KB)

Supplementary file5 (XLSX 35 KB)

Supplementary file6 (DOCX 15 KB)

Supplementary file7 (XLSX 4166 KB)

Supplementary file8 (XLSX 5122 KB)

Supplementary file9 (XLSX 15 KB)

Supplementary file10 (XLSX 57 KB)

Supplementary file11 (XLSX 1191 KB)

## Data Availability

The ac4C-RIP-Seq, NAT10-RIP-Seq, miRNA-Seq and RNA-Seq data generated in this study were provided as EXCEL profiles in Extended Data and have been deposited at the Gene Expression Omnibus (GEO) database (Project id: GSE223148), and the accession link/code for high-throughput sequencing data is available: https://www.ncbi.nlm.nih.gov/geo/query/acc.cgi?acc=GSE223148. All data needed to evaluate the conclusions in the paper are present in the paper and/or the Extended Data Materials, and could also available from the corresponding author (J.Y.) upon reasonable request. Source data are provided with this paper.
