# Supplementary material for: N4-acetylcytidine modifies primary microRNAs for processing in cancer cells
Source: Cell Mol Life Sci. 2024 Feb 3;81(1):73. doi: 10.1007/s00018-023-05107-w (PMC10838262; doi:10.1007/s00018-023-05107-w)
Supplement: Supplementary file 1 — Supplementary file1 (PDF 12798 KB) [file 18_2023_5107_MOESM1_ESM.pdf]

Supplementary material

N4-acetylcytidine modifies primary microRNAs for processing in cancer cells

Hailong Zhang[1, 2#], Runhui Lu[1#], Jiayi Huang[1#], Lian Li[1], Yingting Cao[1], Caihu Huang[1], Ran Chen[1], Yanli Wang[1], Jian Huang[1], Xian Zhao[1, ✉], Jianxiu Yu[1, ✉]

[1] Department of Biochemistry and Molecular Cell Biology, Shanghai Key Laboratory of Tumor Microenvironment and Inflammation, Shanghai Jiao Tong University School of Medicine, Shanghai 200025, China

[2] Institute of Translational Medicine, National Center for Translational Medicine (Shanghai), Shanghai Jiao Tong University, Shanghai 200240, China

[#]Hailong Zhang, Runhui Lu and Jiayi Huang contributed equally.

✉Corresponding authors at: Department of Biochemistry and Molecular Cell Biology, Shanghai Key Laboratory of Tumor Microenvironment and Inflammation, Shanghai Jiao Tong University School of Medicine, Shanghai 200025, China.

E-mail addresses:

Dr. Jianxiu Yu, Jianxiu.Yu@gmail.com or Jianxiu.Yu@shsmu.edu.cn

Dr. Xian Zhao, Xianzhao1985@sjtu.edu.cn

Supplementary Figure Legends

Supplementary Fig. S1 Pri-miRNAs are ac4C modified. **a** 293T cells transiently transfected with HA-DROSHA were treated by UV for CLIP with anti-HA antibody. ac4C-modified RNAs bound to HA-DROSHA were treated with or without RNase A and then conducted by WB with anti-ac4C antibody. **b** Peak density of ac4C summits parsed by location within CDS or UTRs for all ac4C-modified transcripts. **c** IGV tracks displaying examples of sequencing read clusters from two replicates of ac4C-RIP-Seq are shown next to the pre-let-7a-1/pre-let-7f-1 cluster, pre-miR-138-1 and pre-miR-4521 genomic loci.

Supplementary Fig. S2 NAT10 and THUMPD1 interact with and catalyze ac4C modification of pri-miRNAs. **a, f** Structures and lengths of pri-miR-9-1, pri-miR-29b-1 (**a**) and L-for pri-miR-9-1 (**f**) transcription *in vitro*. **b** His-NAT10 and His-THUMPD1 were expressed in *E. coli* BL21(DE3) and purified. **c-d** EMSA assays showing that NAT10 (**c**) and THUMPD1 (**d**) directly interact with pri-miR-9-1. *In vitro* transcribed and biotin-tagged pri-miR-9-1 were co-incubated with purified His-NAT10 or His-THUMPD1, then free pri-miR-9-1 and His-NAT10- or His-THUMPD1-bound pri-miR-9-1 were separated by native 7% polyacrylamide gels and followed by Northern blotting analysis with streptavidin-conjugated HRP. **e** Flag-NAT10 protein was purified from 293T cells ectopically expressing Flag-NAT10 by using 3x Flag peptides. **g** Biotin-tagged 18S-rRNA-45h (50nt), L-pri-miR9-1 (413nt) and pri-miR-29b-1 (144nt) were transcribed by using T7 RNA polymerase,

and subsequently examined by Northern blotting with streptavidin-conjugated HRP. **h** NAT10-binding conserved motifs were identified by DREME (Discriminative Regular Expression Motif Elicitation) with NAT10-RIP-Seq peaks (E-values, the sites were found under this motif out of top 1,000 scored peaks), the conserved nucleotides within these NAT10-recognized motifs were colored in red. **i** Peak density of NAT10-binding summits parsed by location within CDS or UTRs for all transcripts. **j** Density map showing the enrichment of NAT10-RIP-Seq and ac4C-RIP-Seq clusters across all pre-miRNA centers. The x axis represents the distance to the pre-miRNA center, and the y axis represents the normalized density of RIP-Seq clusters. **k** IGV tracks displaying examples of sequencing read clusters from ac4C-RIP-Seq and NAT10-RIP-Seq are shown next to the pre-miR-4747 and pre-miR-1178 genomic loci. **l-m** NAT10 was stably knocked down by shRNA in A549 (**l**) and H1299 (**m**) cell lines. The expression levels of NAT10 were examined by WB and the ac4C levels of 18S rRNA were determined by Northern blot analysis.

Supplementary Fig. S3 Knockdown of NAT10 decreases the expression levels of mature miRNAs. **a** Heatmap of differentially expressed (shNAT10/pLKO.1 >= 1.5 FC) mature miRNAs in A549-shNAT10 stable cell lines. **b** Mean expression levels and differentially expressed (shNAT10/pLKO.1 >= 1.5 FC) of mature miRNAs were analyzed according to miRNA-Seq data in A549-shNAT10 stable cell lines. **c** NAT10 was stably knocked down by shRNA in DU145 cell lines. The expression levels of NAT10 were examined by WB. **d** Knockdown of NAT10 decreases the expression levels of miR-9-5p, miR-29b-3p, let-7a-3p, miR-21-3p and miR-186-5p. The

expression levels of mature miRNAs were determined by qRT-PCR and normalized by U6 snoRNA. Data were mean ± s.d., n > = 3 biologically independent samples, and P-values were calculated by unpaired two-sided t-test.

Supplementary Fig. S4 Pri-miRNA ac4C promotes its processing. **a-b** Flag-tagged NAT10 and THUMPD1 were stably overexpressed in A549 (**a**) and H1299 (**b**)cell lines. **c-d** For non-ac4C-modified pri-miRNAs, pri-miR-9-1 (137 nt) and pri-miR-29b-1 (144 nt) were internally tagged by using Bio-16-UTP as well as ATP, UTP, CTP and GTP through T7 RNA polymerase transcription. And for ac4C-modified pri-miRNAs, pri-miR-9-1 and pri-miR-29b-1 were internally tagged by using Bio-16-UTP as well as ATP, UTP, ac4CTP and GTP through T7 RNA polymerase transcription. These transcribed ac4C-modified and non-ac4C modified pri-miRNAs were purified from 8M Urea/ 15% PAGE gel, subsequently, gradient-increased ac4C-modified and non-ac4C-modified pri-miR-9-1 and pri-miR-29b-1 were analyzed by Northern blotting through anti-ac4C antibody and streptavidin-conjugated HRP, respectively.

Supplementary Fig. S5 Pri-miRNA ac4C promotes its interaction with DGCR8. **a** Lysates from H1299-shNAT10 stable cell lines were immunoprecipitated with anti-DROSHA and normal control IgG antibodies, DGCR8 bound to DROSHA was detected by WB. **b** A schematic illustration of the DGCR8-RIP assay. A549 cells were immunoprecipitated with anti-DGCR8 antibody, then the DGCR8-beads was washed three times with RIP-lysis buffer. Subsequently, in vitro purified pri-miR-9-1,

ac4C-pri-miR-9-1, pri-miR-29b-1 or ac4C-pri-miR-29b-1 were co-incubated with DGCR8-beads. Further, the DGCR8-pri-miRNA-beads were washed three times with RIP-lysis buffer. Finally, small part of the DGCR8-pri-miRNA-beads were conducted for Western blotting analysis to exam IP efficiency, meanwhile pri-miRNAs bound to DGCR8 from the left DGCR8-pri-miRNA-beads were isolated by TRIZOL reagent and determined by Northern blotting analysis. **c** GST-DGCR8 was expressed in BL21(DE3) and purified. **d** The interaction of NAT10 with DGCR8 independent on pri-miRNAs, A549 cells were lysed with RIPA-lysis buffer and following treated with or without RNase A. Then, the cell lysates were immunoprecipitated with anti-NAT10 antibody, DGCR8 associated with NAT10 was determined by western blotting analysis. **e** A schematic illustration of pri-miR-9-1 associate to GST-DGCR8 and NAT10. In brief, GST-DGCR8, His-NAT10 and L-pri-miR-9-1 were co-incubated *in vitro* conditions, and then pulled down by GST beads or RIP with anti-NAT10 antibody, the levels of L-pri-miR-9-1 binding to GST-DGCR8 or NAT10 was extracted and detected by RT-qPCR. **f** L-pri-miR-9-1 directly interact with GST-DGCR8, and NAT10 reduced the interaction of L-pri-miR-9-1 with GST-DGCR8. **g** L-pri-miR-9-1 directly interact with His-NAT10, and GST-DGCR8 decreased the association of L-pri-miR-9-1 with His-NAT10. The pull-down efficiency of GST-DGCR8 (**f**) and His-NAT10 (**g**) were analyzed by WB, L-pri-miR-9-1 associated with GST-DGCR8 and His-NAT10 were determined by qPCR. Data were mean ± s.d., n > = 3 biologically independent samples, and P-values were calculated by unpaired two-sided t-test. **h** Flag-DROSHA protein was purified

from 293T cells ectopically expressing Flag-DROSHA by using 3x Flag peptides. **i-j** ac4C-pri-miR-9-1 and ac4C-pri-miR-29b-1 interacted with only DGCR8, but not with DROSHA. Biotin-tagged ac4C-pri-miR-9-1 (**i**) and ac4C-pri-miR-29b-1 (**j**) were co-incubated with GST-DGCR8 or Flag-DGCR8 for *in vitro* EMSA assays. Free ac4c-pri-miRNAs and protein/ac4c-pri-miRNA complex were separated on native 7% polyacrylamide gels, and followed by Northern blotting analysis with streptavidin-conjugated HRP. **k** A proposed model that ac4C modification of pri-miRNAs by NAT10/THUMPD1 facilitates its affinity to DGCR8 for following processing. Microprocessor DGCR8/DROSHA and NAT10/THUMPD1 were recruited to different pri-miRNA transcripts which transcribed by RNA pol II/III. Pri-miRNAs binding to microprocessor were processed into mature miRNAs. Whereas, a subset of pri-miRNAs were firstly interacted with NAT10/THUMPD1 for following ac4C modification, then ac4C augments pri-miRNAs affinity to DGCR8, and finally promotes pri-miRNA processing into mature miRNA

Supplementary Fig. S6 a The expression levels of NAT10 mRNA in several tumor types from TCGA database, and there was no significant difference ($p>0.05$) in the expression levels of NAT10 mRNA between tumor tissues and normal tissues. **b** Bar plot showing the percentage of patients with mutations in NAT10 across multiple cancer types.

Supplementary Fig. S7 a Knockdown of NAT10 augmented the expression levels of miR-9-5p targets, including SH3BP4, NCOR2, LMNA, EPAS1 and TES. The

expression levels of indicated mRNAs were performed by qRT-PCR in A549-shNAT10 cell lines. qRT-PCR data were mean ± s.d., n > = 3 biologically independent samples, and P-values were calculated by unpaired two-sided t-test. **b** Knockdown of NAT10 increased the accumulation of mRNA transcripts (mean FPKM >= 30) targeted by miR-29b-3p (*P*=0.0640). **c** Mature miR-29b-3p was highly expressed in lung cancer tissues. Analysis of the expression levels of miR-29b-3p between normal tissues (n = 91) and lung cancer tissues (n = 999) from TCGA lung cancer miRNA-Seq data (*P*-value < 0.0001). Data were mean ± s.d., *P*-values were determined by unpaired two-sided t-test. **d** The expression levels of NAT10 were negatively correlated with pri-miR-29b-1 (Pearson *r* = -0.8135, *P*-value (two tailed) < 0.0001) from TCGA-Lung cancer (n = 1028) RNA-Seq data. Pearson's correlation was analyzed by using GraphPad Prism 8. **e** The Spearman's correlation of mRNA expression levels in human TCGA lung normal and TCGA lung cancer tissues between NAT10 with DGCR8, DROSHA, XPO5, DICER1, AGO2 or TARBP2, respectively (R, Spearman correlation coefficient). **f** Heatmap showing the Spearman's correlation and estimated significance of DGCR8, DROSHA, XPO5, DICER1, AGO2 or TARBP2 with NAT10 mRNA levels across all TCGA cancer types.

Supplementary Table S1 Primer or oligonucleotide sequences used in the experiments.

Supplementary Table S2 ac4C-RIP-seq data (mRNA part) in A549 cell line.

Supplementary Table S3 ac4C-RIP-seq data (LncRNA part) in A549 cell line.

Supplementary Table S4 ac4C-RIP-seq data (pri-miRNA part) in A549 cell line.

Supplementary Table S5 Prediction of ac4C (N4-acetylcytidine) modification sites of pri-miR9-1 by using PACES.

Supplementary Table S6 NAT10-RIP-seq data (mRNA part) in A549 cell line.

Supplementary Table S7 NAT10-RIP-seq data (LncRNA part) in A549 cell line.

Supplementary Table S8 NAT10-RIP-seq data (pri-miRNA part) in A549 cell line.

Supplementary Table S9 miRNA-seq data in A549-shNAT10 stable cell lines.

Supplementary Table S10 RNA-seq data in A549-shNAT10 stable cell lines.

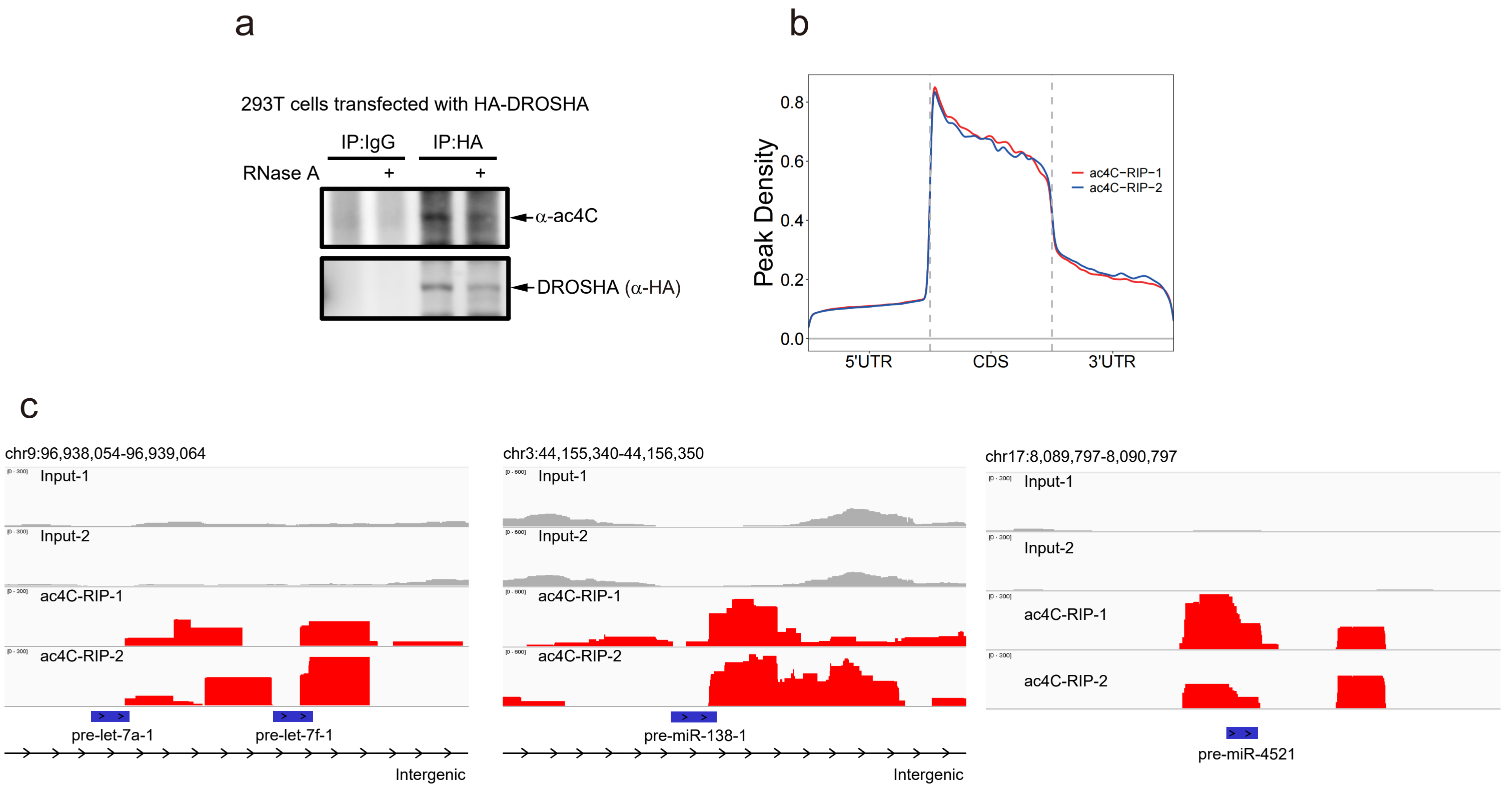
a
293T cells transfected with HA-DROSHA
IP:IgG
IP:HA
RNase A
+
+
α-ac4C
DROSHA (α-HA)
b
Peak Density
0.8
0.6
0.4
0.2
0.0
ac4C-RIP-1
ac4C-RIP-2
5'UTR
CDS
3'UTR
c
chr9:96,938,054-96,939,064
Input-1
Input-2
ac4C-RIP-1
ac4C-RIP-2
pre-let-7a-1
pre-let-7f-1
Intergenic
chr3:44,155,340-44,156,350
Input-1
Input-2
ac4C-RIP-1
ac4C-RIP-2
pre-miR-138-1
Intergenic
chr17:8,089,797-8,090,797
Input-1
Input-2
ac4C-RIP-1
ac4C-RIP-2
pre-miR-4521

Zhang_Fig. S2

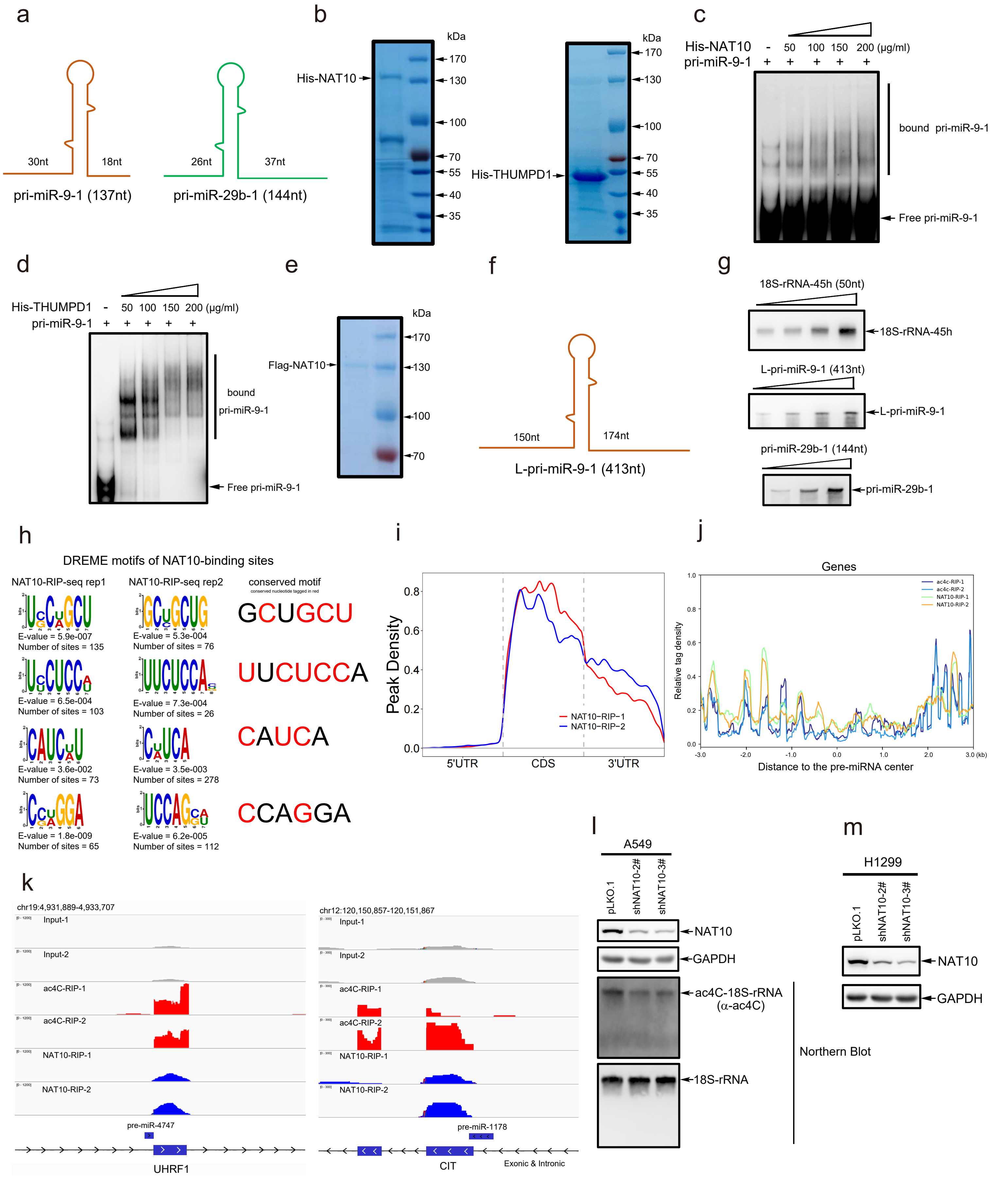
a
30nt
18nt
pri-miR-9-1 (137nt)
26nt
37nt
pri-miR-29b-1 (144nt)
b
His-NAT10
kDa
170
130
100
70
55
40
35
His-THUMPD1
c
His-NAT10 - 50 100 150 200 (μg/ml)
pri-miR-9-1 + + + + +
bound pri-miR-9-1
Free pri-miR-9-1
d
His-THUMPD1 - 50 100 150 200 (μg/ml)
pri-miR-9-1 + + + + +
bound
pri-miR-9-1
Free pri-miR-9-1
e
Flag-NAT10
kDa
170
130
100
70
f
150nt
174nt
L-pri-miR-9-1 (413nt)
g
18S-rRNA-45h (50nt)
18S-rRNA-45h
L-pri-miR-9-1 (413nt)
L-pri-miR-9-1
pri-miR-29b-1 (144nt)
pri-miR-29b-1
h
DREME motifs of NAT10-binding sites
NAT10-RIP-seq rep1
NAT10-RIP-seq rep2
conserved motif
E-value = 5.9e-007
Number of sites = 135
E-value = 5.3e-004
Number of sites = 76
GCUGCU
E-value = 6.5e-004
Number of sites = 103
E-value = 7.3e-004
Number of sites = 26
UUCUCCA
E-value = 3.6e-002
Number of sites = 73
E-value = 3.5e-003
Number of sites = 278
CAUCA
E-value = 1.8e-009
Number of sites = 65
E-value = 6.2e-005
Number of sites = 112
CCAGGA
i
Peak Density
0.0
0.2
0.4
0.6
0.8
NAT10-RIP-1
NAT10-RIP-2
5'UTR
CDS
3'UTR
j
Genes
Relative tag density
ac4c-RIP-1
ac4c-RIP-2
NAT10-RIP-1
NAT10-RIP-2
Distance to the pre-miRNA center
k
chr19:4,931,889-4,933,707
Input-1
Input-2
ac4C-RIP-1
ac4C-RIP-2
NAT10-RIP-1
NAT10-RIP-2
pre-miR-4747
UHRF1
chr12:120,150,857-120,151,867
Input-1
Input-2
ac4C-RIP-1
ac4C-RIP-2
NAT10-RIP-1
NAT10-RIP-2
pre-miR-1178
CIT
Exonic & Intronic
l
A549
pLKO.1
shNAT10-2#
shNAT10-3#
NAT10
GAPDH
ac4C-18S-rRNA
(α-ac4C)
18S-rRNA
Northern Blot
m
H1299
pLKO.1
shNAT10-2#
shNAT10-3#
NAT10
GAPDH

Zhang_Fig. S3

a

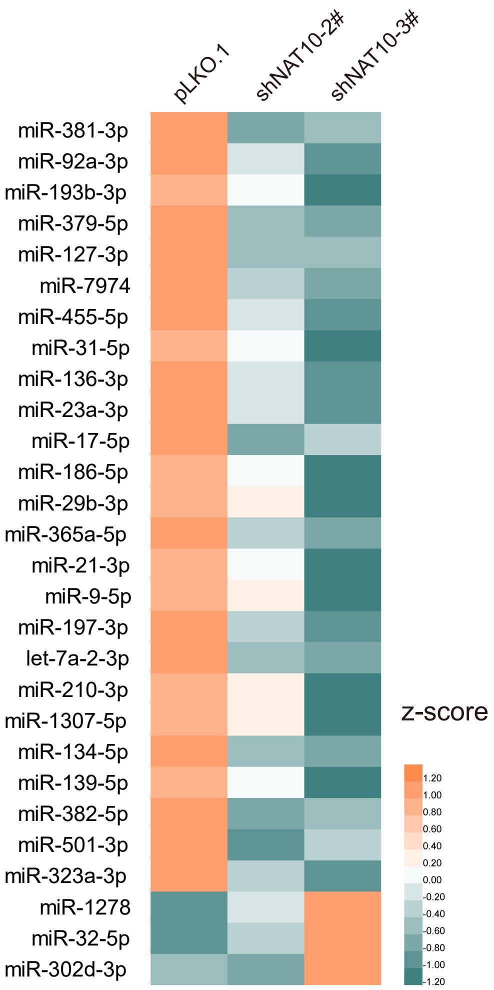

b

	miRNAs	Mean log2 (TPM+1)	Log2 FC (shNAT10/pLKO.1)
1	hsa-miR-381-3p	9.483	-0.604
2	hsa-miR-92a-3p	8.006	-0.643
3	hsa-miR-193b-3p	7.582	-1.015
4	hsa-miR-379-5p	7.668	-0.639
5	hsa-miR-127-3p	7.334	-0.604
6	hsa-miR-7974	6.658	-0.704
7	hsa-miR-455-5p	5.700	-1.111
8	hsa-miR-31-5p	5.799	-0.879
9	hsa-miR-136-3p	5.575	-0.696
10	hsa-miR-23a-3p	5.544	-0.585
11	hsa-miR-17-5p	5.358	-0.741
12	hsa-miR-186-5p	5.248	-0.797
13	hsa-miR-29b-3p	5.298	-0.706
14	hsa-miR-365a-5p	5.000	-0.637
15	hsa-miR-21-3p	4.891	-0.642
16	hsa-miR-9-5p	4.790	-0.756
17	hsa-miR-197-3p	4.273	-1.000
18	hsa-let-7a-2-3p	4.322	-0.710
19	hsa-miR-210-3p	4.322	-0.613
20	hsa-miR-1307-5p	4.087	-0.601
21	hsa-miR-134-5p	3.663	-1.152
22	hsa-miR-139-5p	3.807	-0.724
23	hsa-miR-382-5p	3.503	-1.000
24	hsa-miR-501-3p	3.700	-0.628
25	hsa-miR-323a-3p	3.459	-0.737
26	hsa-miR-1278	4.703	0.585
27	hsa-miR-32-5p	3.930	0.750
28	hsa-miR-302d-3p	3.606	2.668

c

DU145 stable cell lines

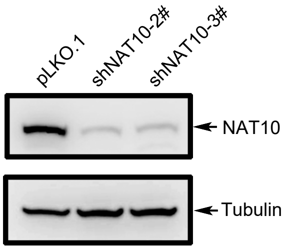

d

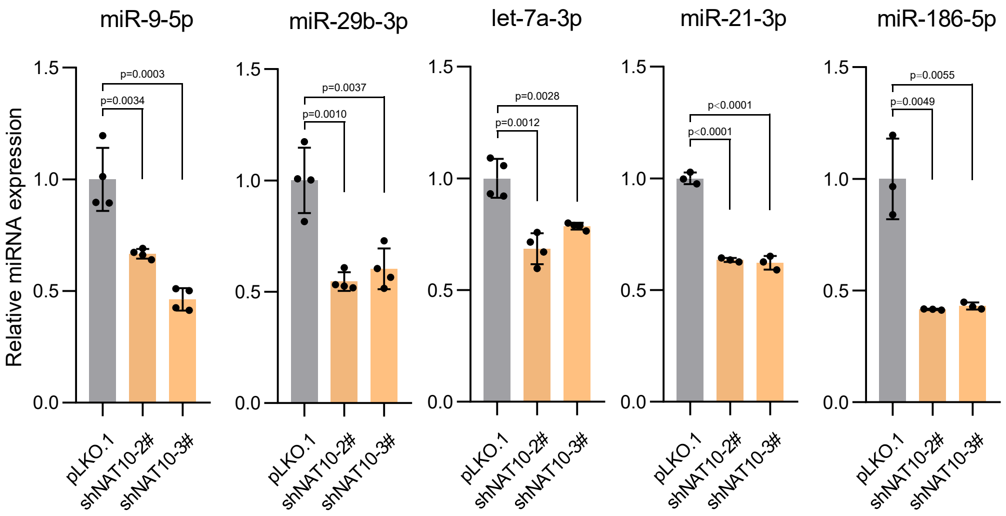

Zhang_Fig. S4

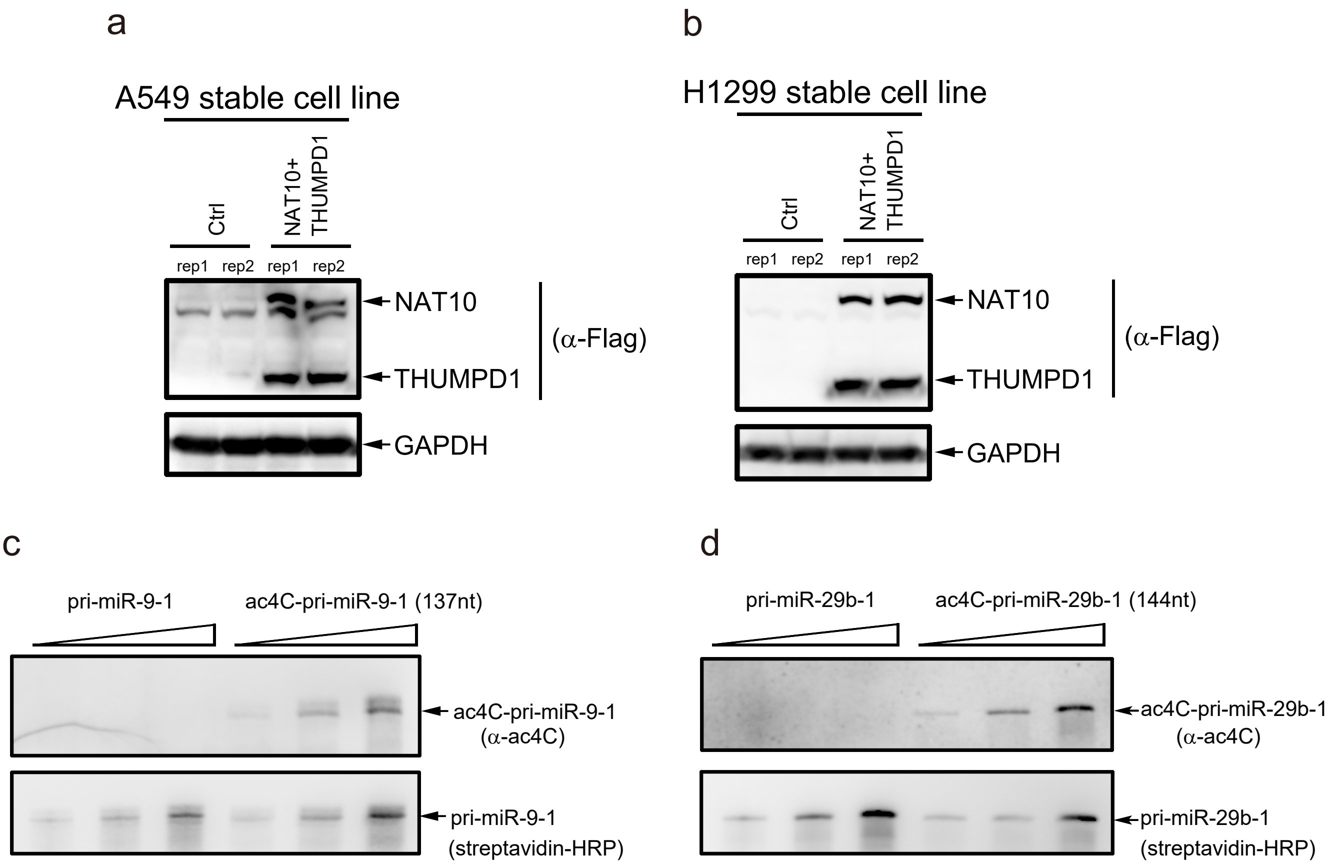
a
A549 stable cell line
Ctrl
NAT10+ THUMPD1
rep1 rep2 rep1 rep2
NAT10
THUMPD1
(α-Flag)
GAPDH
b
H1299 stable cell line
Ctrl
NAT10+ THUMPD1
rep1 rep2 rep1 rep2
NAT10
THUMPD1
(α-Flag)
GAPDH
c
pri-miR-9-1
ac4C-pri-miR-9-1 (137nt)
ac4C-pri-miR-9-1 (α-ac4C)
pri-miR-9-1 (streptavidin-HRP)
d
pri-miR-29b-1
ac4C-pri-miR-29b-1 (144nt)
ac4C-pri-miR-29b-1 (α-ac4C)
pri-miR-29b-1 (streptavidin-HRP)

Zhang_Fig. S5

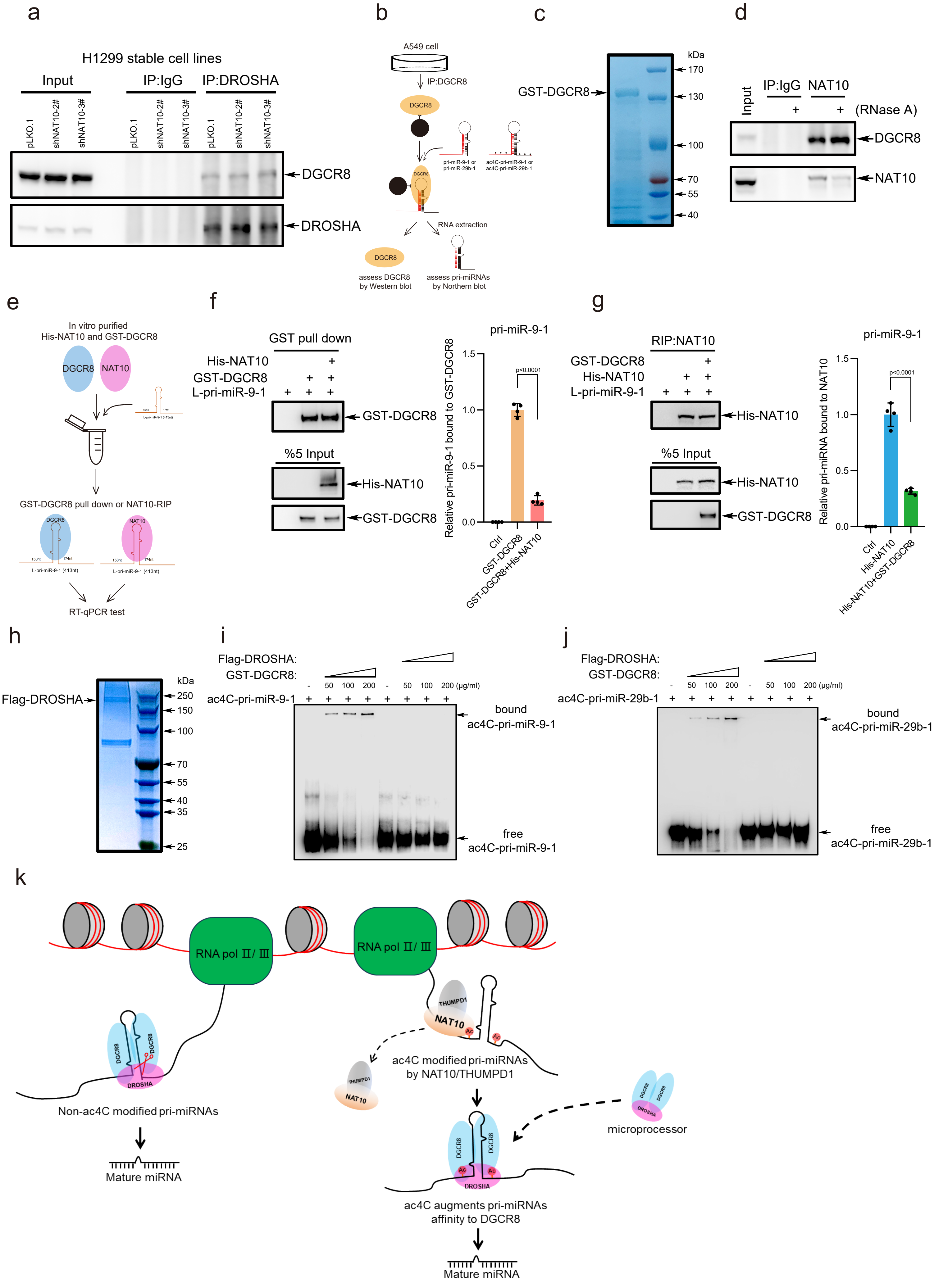
a
H1299 stable cell lines
Input
IP:IgG
IP:DROSHA
pLKO.1
shNAT10-2#
shNAT10-3#
DGCR8
DROSHA
b
A549 cell
IP:DGCR8
DGCR8
pri-miR-9-1 or pri-miR-29b-1
ac4C-pri-miR-9-1 or ac4C-pri-miR-29b-1
RNA extraction
assess DGCR8 by Western blot
assess pri-miRNAs by Northern blot
c
kDa
GST-DGCR8
170
130
100
70
55
40
d
Input
IP:IgG
NAT10
(RNase A)
DGCR8
NAT10
e
In vitro purified His-NAT10 and GST-DGCR8
DGCR8
NAT10
GST-DGCR8 pull down or NAT10-RIP
L-pri-miR-9-1 (413nt)
RT-qPCR test
f
GST pull down
His-NAT10
GST-DGCR8
L-pri-miR-9-1
GST-DGCR8
%5 Input
His-NAT10
GST-DGCR8
pri-miR-9-1
Relative pri-miR-9-1 bound to GST-DGCR8
p<0.0001
Ctrl
GST-DGCR8
GST-DGCR8+His-NAT10
g
RIP:NAT10
GST-DGCR8
His-NAT10
L-pri-miR-9-1
His-NAT10
%5 Input
His-NAT10
GST-DGCR8
pri-miR-9-1
Relative pri-miRNA bound to NAT10
p<0.0001
Ctrl
His-NAT10
His-NAT10+GST-DGCR8
h
Flag-DROSHA
kDa
250
150
100
70
55
40
35
25
i
Flag-DROSHA:
GST-DGCR8:
- 50 100 200 - 50 100 200 (μg/ml)
ac4C-pri-miR-9-1
bound ac4C-pri-miR-9-1
free ac4C-pri-miR-9-1
j
Flag-DROSHA:
GST-DGCR8:
- 50 100 200 - 50 100 200 (μg/ml)
ac4C-pri-miR-29b-1
bound ac4C-pri-miR-29b-1
free ac4C-pri-miR-29b-1
k
RNA pol Ⅱ/ Ⅲ
THUMPD1
NAT10
DGCR8
DROSHA
Non-ac4C modified pri-miRNAs
Mature miRNA
ac4C modified pri-miRNAs by NAT10/THUMPD1
microprocessor
ac4C augments pri-miRNAs affinity to DGCR8
Mature miRNA

a

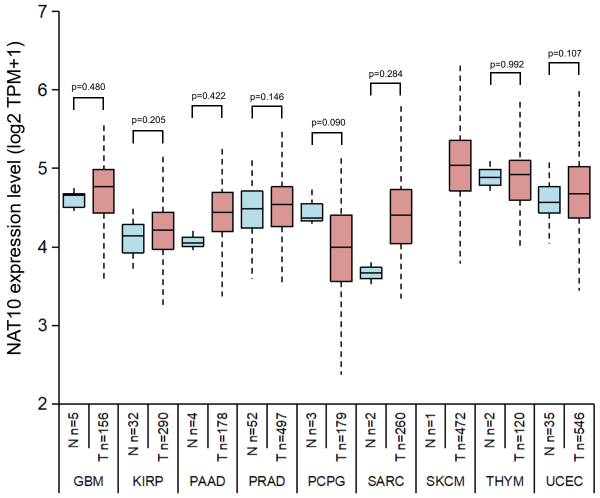
NAT10 expression level (log2 TPM+1)
7
6
5
4
3
2
p=0.480
p=0.205
p=0.422
p=0.146
p=0.090
p=0.284
p=0.992
p=0.107
N n=5
T n=156
N n=32
T n=290
N n=4
T n=178
N n=52
T n=497
N n=3
T n=179
N n=2
T n=260
N n=1
T n=472
N n=2
T n=120
N n=35
T n=546
GBM
KIRP
PAAD
PRAD
PCPG
SARC
SKCM
THYM
UCEC

b

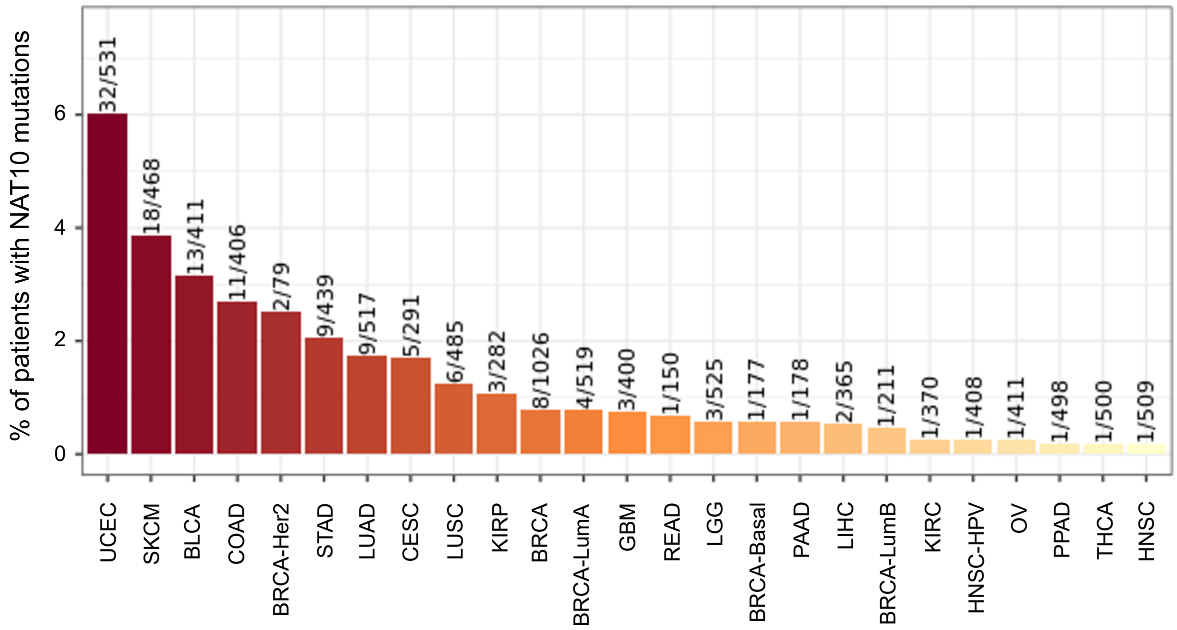
% of patients with NAT10 mutations
6
4
2
0
32/531
18/468
13/411
11/406
2/79
9/439
9/517
5/291
6/485
3/282
8/1026
4/519
3/400
1/150
3/525
1/177
1/178
2/365
1/211
1/370
1/408
1/411
1/498
1/500
1/509
UCEC
SKCM
BLCA
COAD
BRCA-Her2
STAD
LUAD
CESC
LUSC
KIRP
BRCA
BRCA-LumA
GBM
READ
LGG
BRCA-Basal
PAAD
LIHC
BRCA-LumB
KIRC
HNSC-HPV
OV
PPAD
THCA
HNSC

Zhang_Fig. S7

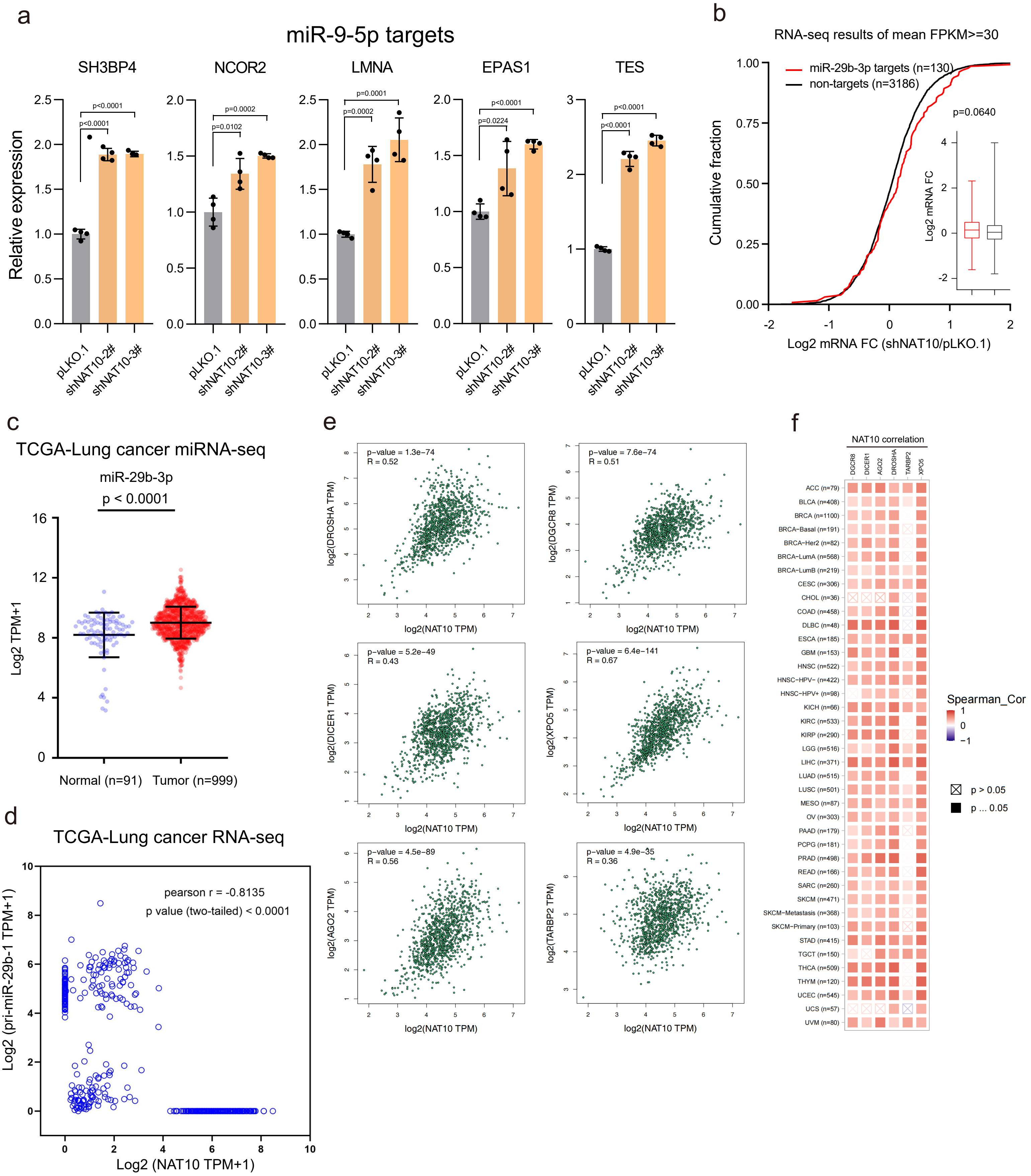
a
miR-9-5p targets
SH3BP4
NCOR2
LMNA
EPAS1
TES
Relative expression
p<0.0001
p<0.0001
p=0.0002
p=0.0102
p=0.0001
p=0.0002
p<0.0001
p=0.0224
p<0.0001
p<0.0001
pLKO.1
shNAT10-2#
shNAT10-3#
b
RNA-seq results of mean FPKM>=30
miR-29b-3p targets (n=130)
non-targets (n=3186)
p=0.0640
Cumulative fraction
Log2 mRNA FC
Log2 mRNA FC (shNAT10/pLKO.1)
c
TCGA-Lung cancer miRNA-seq
miR-29b-3p
p < 0.0001
Log2 TPM+1
Normal (n=91)
Tumor (n=999)
d
TCGA-Lung cancer RNA-seq
pearson r = -0.8135
p value (two-tailed) < 0.0001
Log2 (pri-miR-29b-1 TPM+1)
Log2 (NAT10 TPM+1)
e
p−value = 1.3e−74
R = 0.52
log2(DROSHA TPM)
p−value = 7.6e−74
R = 0.51
log2(DGCR8 TPM)
p−value = 5.2e−49
R = 0.43
log2(DICER1 TPM)
p−value = 6.4e−141
R = 0.67
log2(XPO5 TPM)
p−value = 4.5e−89
R = 0.56
log2(AGO2 TPM)
p−value = 4.9e−35
R = 0.36
log2(TARBP2 TPM)
log2(NAT10 TPM)
f
NAT10 correlation
DGCR8
DICER1
AGO2
DROSHA
TARBP2
XPO5
ACC (n=79)
BLCA (n=408)
BRCA (n=1100)
BRCA−Basal (n=191)
BRCA−Her2 (n=82)
BRCA−LumA (n=568)
BRCA−LumB (n=219)
CESC (n=306)
CHOL (n=36)
COAD (n=458)
DLBC (n=48)
ESCA (n=185)
GBM (n=153)
HNSC (n=522)
HNSC−HPV− (n=422)
HNSC−HPV+ (n=98)
KICH (n=66)
KIRC (n=533)
KIRP (n=290)
LGG (n=516)
LIHC (n=371)
LUAD (n=515)
LUSC (n=501)
MESO (n=87)
OV (n=303)
PAAD (n=179)
PCPG (n=181)
PRAD (n=498)
READ (n=166)
SARC (n=260)
SKCM (n=471)
SKCM−Metastasis (n=368)
SKCM−Primary (n=103)
STAD (n=415)
TGCT (n=150)
THCA (n=509)
THYM (n=120)
UCEC (n=545)
UCS (n=57)
UVM (n=80)
Spearman_Cor
p > 0.05
p ... 0.05
