# Supplementary material for: N4-acetylcytidine modifies primary microRNAs for processing in cancer cells
Source: Cell Mol Life Sci. 2024 Feb 3;81(1):73. doi: 10.1007/s00018-023-05107-w (PMC10838262; doi:10.1007/s00018-023-05107-w)
Supplement: Supplementary file 2 — Supplementary file2 (DOCX 20 KB) [file 18_2023_5107_MOESM2_ESM.docx]

**Supplementary Table S1** Primer or oligonucleotide sequences were used in this study.

| The sequences of plasmids construction, shRNA and pri-miRNA primers for QRT-PCR | | |
| --- | --- | --- |
| plasmids construction | | |
| plasmids | Primer | Sequence（5’-3’） |
| Flag-NAT10 | Forward | CCGAATTC ATGCATCGGAAAAAGGTGGATAAC |
|  | Reverse | ATAAGAATGCGGCCGC CTATTTCTTCCGCTTCAGTTTC |
| Flag-THUMPD1 | Forward | CGGAATTCATGGCGGCCCCTGCCCAGCAGAC |
|  | Reverse | ATAAGAATGCGGCCGC CTATGAGAAGTCATTTTCATTTG |
| T7-RNA polymerase promoter linked RNAs primers | | |
| T7-RNA polymerase promoter-Long-pri-miR-9-1 (413nt) | Forward | TTAATACGACTCACTATAGGGAGAACTTCATTTCTCTCTTCACCCTC |
|  | Reverse | CTACCTGTGCCGACTGGGGTG |
| T7-RNA polymerase promoter-pri-miR-9-1 (137nt) | Forward | TTAATACGACTCACTATAGGGAGAGTGGAAGAGGCGGCGACAGCAG |
|  | Reverse | GCCCCTCTGCGCAGTGTATGG |
| T7-RNA polymerase promoter-pri-miR-29b-1 | Forward | TTAATACGACTCACTATAGGGAGA GAAGAAAAAAATAGATCATAA |
|  | Reverse | ACTTCTTTTGCTGTTGGTAGT |
| T7-RNA polymerase promoter-18S-rRNA-45h | Forward | TTAATACGACTCACTATAGGGAGA GTAAAAGTCGTAACAAGGTTTC |
|  | Reverse | TAATGATCCTTCCGCAGGTTC |
| shRNA sequences | | |
| NAT10 shRNA-1 | Forward | CCGGTCAGCACCACTGCTGAGAATAAGACTCGAGTCTTATTCTCAGCAGTGGTGCTGTTTTTG |
|  | Reverse | AATTCAAAAACAGCACCACTGCTGAGAATAAGACTCGAGTCTTATTCTCAGCAGTGGTGCTGA |
| NAT10 shRNA-2 | Forward | CCGGTAGAACCCAAACAGAGCAAGAAGTTGCTCGAGCAACTTCTTGCTCTGTTTGGGTTCTTTTTTG |
|  | Reverse | AATTCAAAAAAGAACCCAAACAGAGCAAGAAGTTGCTCGAGCAACTTCTTGCTCTGTTTGGGTTCTA |
| NAT10 shRNA-3 | Forward | CCGGTCCTGTAGTTTATGTAGAATGCCACACTCGAGTGTGGCATTCTACATAAACTACAGGTTTTTG |
|  | Reverse | AATTCAAAAACCTGTAGTTTATGTAGAATGCCACACTCGAGTGTGGCATTCTACATAAACTACAGGA |
| QRT-PCR primers | | |
| pri-miR-381 | Forward | TGGTACTTAAAGCGAGGTTGC |
|  | Reverse | GGTCATGCACACACATACCAC |
| pri-miR-92a-1 | Forward | CTGTGTGATATTCTGCTGTGC |
|  | Reverse | ATCTTCTGGTCACAATCCCC |
| pri-miR-193b | Forward | AATGGGGACTCACTTCTTGG |
|  | Reverse | AAACTCATCTCGCCCTCAAA |
| pri-miR-379 | Forward | TGGGGTCAGCACCATTCCGTG |
|  | Reverse | AGCCCTCCGAGGATGGATTGG |
| pri-miR-186 | Forward | GCACCACAGCCTGGGGAACAG |
|  | Reverse | TGATGGGTGTTCTGTAAACCT |
| pri-miR-29b-1 | Forward | TTCAGGAAGCTGGTTTCATA |
|  | Reverse | TGGCAGGGGTGAGGAAGGGG |
| pri-miR-21 | Forward | TTTTGTTTTGCTTGGGAGGA |
|  | Reverse | AGCAGACAGTCAGGCAGGAT |
| pri-miR-9-1 | Forward | GAAGCTGCGGAGGTGCTGGC |
|  | Reverse | GGCCCCTCTGCGCAGTGTAT |
| pri-miR-197 | Forward | GCAGTCCCTGGCCCAACACC |
|  | Reverse | ATCCCCACGGTGAGACATAAC |
| pri-miR-210 | Forward | GAGGGATCCCAGGTTGGCGG |
|  | Reverse | CACGCACAGTGGGTCTGGGG |
| pri-let-7a-2 | Forward | GGCCCAAATAGGTGACAGCAC |
|  | Reverse | ACCCAAGGAAAGCTAGGAGGC |
| *β-Actin* | Forward | GCACAGAGCCTCGCCTT |
|  | Reverse | GTTGTCGACGACGAGCG |
| *GAPDH* | Forward | CTCAAGGGCATCCTGGGCTA |
|  | Reverse | ATGAGGTCCACCACCCTGTT |
| *SH3BP4* | Forward | ACCCTGATTGACCTGAGCGA |
|  | Reverse | GGGGTTGTCTACGAGCAAGG |
| *NCOR2* | Forward | CACGAGGTGTCAGAGATCATCG |
|  | Reverse | GCCATAAGCCCGTTCATGTTG |
| *LMNA* | Forward | AATGATCGCTTGGCGGTCTAC |
|  | Reverse | CACCTCTTCAGACTCGGTGAT |
| *EPAS1* | Forward | CGGAGGTGTTCTATGAGCTGG |
|  | Reverse | AGCTTGTGTGTTCGCAGGAA |
| *TES* | Forward | AGTGCCATGAGTTGTCTCCC |
|  | Reverse | GGGTGCTTCTATCCCCTCCA |
| miR-9-5p-RT | Reverse | GTCGTATCCAGTGCAGGGTCCGAGGTATTCGCACTGGATACGACTCATAC |
| miR-9-5p-QRT | Forward | GCCTGTCTTTGGTTATCTAGC |
| miR-29b-3p-RT | Reverse | GTCGTATCCAGTGCAGGGTCCGAGGTATTCGCACTGGATACGACAACACT |
| miR-29b-3p-QRT | Forward | GCCTGTAGCACCATTTGAAA |
| let-7a-3p-RT | Reverse | GTCGTATCCAGTGCAGGGTCCGAGGTATTCGCACTGGATACGACGAAAGA |
| let-7a-3p-QRT | Forward | GCCTGCTATACAATCTACTG |
| miR-21-3p-RT | Reverse | GTCGTATCCAGTGCAGGGTCCGAGGTATTCGCACTGGATACGACACAGCC |
| miR-21-3p-QRT | Forward | GCCTGCAACACCAGTCGATG |
| miR-186-5p-RT | Reverse | GTCGTATCCAGTGCAGGGTCCGAGGTATTCGCACTGGATACGACAGCCCA |
| miR-186-5p-QRT | Forward | GCCTGCAAAGAATTCTCCTT |
| mature-miRNA-common-QRT | Reverse | GTGCAGGGTCCGAGGT |
| U6-QRT | Forward | CGCTTCGGCAGCACATATAC |
| U6-QRT | Reverse | AGGGGCCATGCTAATCTTCT |
| Northern blot probe sequences | | |
| miR-21 | sense | AACTATACAACCTACTACCTCA |
| let-7a | sense | TCAACATCAGTCTGATAAGCTA |
| miR-9 | sense | AACACTGATTTCAAATGGTGCTA |
| miR-29b | sense | TCATACAGCTAGATAACCAAAGA |
| U6 | sense | TGTGCTGCCGAAGCGAGCAC |
