# Supplementary material for: N4-acetylcytidine modifies primary microRNAs for processing in cancer cells
Source: Cell Mol Life Sci. 2024 Feb 3;81(1):73. doi: 10.1007/s00018-023-05107-w (PMC10838262; doi:10.1007/s00018-023-05107-w)
Supplement: Supplementary file 6 — Supplementary file6 (DOCX 15 KB) [file 18_2023_5107_MOESM6_ESM.docx]

**Supplementary Table S5** Prediction of ac4C (N4-acetylcytidine) modification sites of pri-miR9-1 by using PACES (http://www.rnanut.net/paces/).

| **Sequence of pri-miR9-1**  **(500-bp flanking sequences of known pre-miR9-1 (miRBase, tagged in blue) was extracted as pri-miR9-1)** | **Prediction of ac4C sequences** | **start** | **end** | **Score**  **thresholds:** |
| --- | --- | --- | --- | --- |
| AGGCTCGGGCTCCGCCGAAGGCAGGGCTCTGGCTGTCGGATGCGCCCTCGATCTTCCAGAGAAGGGCAGTGGAGACCCGGCGAGGCTGGGGAGGCCTCTGCCTGGGTCGCTGCGGTCTTCCCGGGTGGCACGAAAAGCCCGCGCGTCCCTTCCACCTGGGACCAGCGGAACTCCTGCTCCTGGCCCGAATGGGAGAGCGGGAAATGGGGCACCAGAAACTTTTTGGGTCTGGATCGGGGTCAACTCCCCGACTTCGACGCCACGGGTCATCGCGTCCTTTCCACGCCTTTTTCGGTCTCTGTCGTGTCTGTATCTCCGTGTCTGAGGTTTTTGTTGTTGTTTTGTCTCGGACTTCATTTCTCTCTTCACCCTCCCCCTCAACTCCACTCGTGTCCCTTCCCTCCTACTCCCGCTGACGGGCGACCGGGAAGCTGCGGAGGTGCTGGCGGCGGCGGCGGCCGGGAGGCTGCGTGGAAGAGGCGGCGACAGCAGCCAGGAGG**CGGGGTTGGTTGTTATCTTTGGTTATCTAGCTGTATGAGTGGTGTGGAGTCTTCATAAAGCTAGATAACCGAAAGTAAAAATAACCCCA**TACACTGCGCAGAGGGGCCCGGGGAAAGCCGGCCTCACGGGCGGGAGGGAGAGGCTTGAGCGAGGGGCTAGAGCCGCGAGGACTGACCAGCACCAGAGGATACAAGAGGAGGAGAGAGACCTCACTGGTTCGGGCGACATTTCACCCCCCCAGCACCCCAGTCGGCACAGGTAGGAGTCACAGTCCCATTTCACTTCAGCCAGGTGCCATATTTTTCCCCCAGCAGAGTTGAGGGGGAGCAAGGAAGCCTCATTGTGGTGCCCTCTGGTGGTCCCCGCTCAGGATGGGGCCTTTTCTGGCAGTCTGGGTCACTGCTTGGGAAGCTGATCTAAGGGAGAAATCAGTTGAAATCTAGCTGCAGGGTTATTCCGCCTCCCGGCCCAGGCCTCTCCCTCCAGCGGGAGCTGGTGAACACTTTCCCACAGGCTGTCCACAGCTATGGCTGGGTGGGGGAGTTTGGAGGAAGTCACCTGAGAACACTGGTCTTTGCTTAAGTCGGC | **CGACTTCGACGCCAC** | **249** | **263** | **0.2740** |
|  | **CCCCCTCAACTCCAC** | **373** | **387** | **0.2065** |
|  | **CGGCGACAGCAGCCA** | **481** | **495** | **0.2214** |
|  | **CCCCCCCAGCACCCC** | **734** | **748** | **0.2592** |
